# Metabolome-Wide Associations of Gestational Weight Gain in Pregnant Women with Overweight and Obesity

**DOI:** 10.3390/metabo12100960

**Published:** 2022-10-11

**Authors:** Jin Dai, Nansi S. Boghossian, Mark A. Sarzynski, Feng Luo, Xiaoqian Sun, Jian Li, Oliver Fiehn, Jihong Liu, Liwei Chen

**Affiliations:** 1 Department of Epidemiology, Fielding School of Public Health, University of California, Los Angeles, CA 90095, USA; 2 Department of Epidemiology and Biostatistics, Arnold School of Public Health, University of South Carolina, Columbia, SC 29208, USA; 3 Department of Exercise Science, Arnold School of Public Health, University of South Carolina, Columbia, SC 29208, USA; 4 School of Computing, Clemson University, Clemson, SC 29634, USA; 5 Department of Mathematical and Statistical Sciences, Clemson University, Clemson, SC 29634, USA; 6 Department of Environmental Health Sciences, Fielding School of Public Health, University of California, Los Angeles, CA 90095, USA; 7 School of Nursing, University of California, Los Angeles, CA 90095, USA; 8 West Coast Metabolomics Center, University of California, Davis, CA 95616, USA

**Keywords:** biomarkers, gestational weight gain, metabolomics, pregnancy, obesity, overweight

## Abstract

Excessive gestational weight gain (GWG) is associated with adverse pregnancy outcomes. This metabolome-wide association study aimed to identify metabolomic markers for GWG. This longitudinal study included 39 Black and White pregnant women with a prepregnancy body mass index (BMI) of ≥ 25 kg/m^2^. Untargeted metabolomic profiling was performed using fasting plasma samples collected at baseline (mean: 12.1 weeks) and 32 weeks of gestation. The associations of metabolites at each time point and changes between the two time points with GWG were examined by linear and least absolute shrinkage and selection operator (LASSO) regression analyses. Pearson correlations between the identified metabolites and cardiometabolic biomarkers were examined. Of the 769 annotated metabolites, 88 metabolites at 32 weeks were individually associated with GWG, with four (phosphatidylcholine (PC) 34:4, triacylglycerol (TAG) 52:6, arachidonic acid, isoleucine) jointly associated with GWG (area under the receiver operating characteristic curve (AUC) for excessive GWG: 0.80, 95% CI: 0.67, 0.93). No correlations were observed between the 88 metabolites and insulin, C-peptide, and high-sensitivity C-reactive protein at 32 weeks. Twelve metabolites at baseline (AUC for excessive GWG: 0.80, 95% CI: 0.62, 0.99) and three metabolite changes (AUC for excessive GWG: 0.73, 95% CI: 0.44, 1.00) were jointly associated with GWG. We identified novel metabolites in the first and third trimesters associated with GWG, which may shed light on the pathophysiology of GWG.

## 1. Introduction

Gestational weight gain (GWG), the weight gained during pregnancy, is critical for the health outcomes of mothers and their offspring both immediately and long-term. Excessive GWG is associated with gestational diabetes, gestational hypertensive disorder, postpartum weight retention, and future risk of obesity for mothers, and being large for their gestational age, childhood obesity, and glycemic dysfunction for offspring [1]. To achieve optimal health, the Institute of Medicine (IOM) has recommended GWG guidelines based on the maternal prepregnancy body mass index (BMI) [2]. Unfortunately, the most recent study using national representative samples showed that more than two-thirds of pregnant women in the United States (U.S.) failed to achieve the optimal GWG (i.e., 20.9% had inadequate GWG and 47.2% had excessive GWG) [3], indicating a significant public health problem. Although the prevalence of inadequate GWG has remained stable, the prevalence of excessive GWG has increased from 43% in 2000 to 46% in 2009 in the U.S. [4], likely driven by the current obesity epidemic, unhealthy dietary intake, and inadequate physical activity among women of reproductive ages [4,5,6].

Given the profound health consequences of GWG, there is an urgent need to identify molecular biomarkers and underpinnings of GWG. Pregnancy can result in a series of dynamic physiological changes, including alternations of the maternal hormonal profile, basal metabolic rate, energy storage, partition, and placental metabolism, which are likely related to GWG [7]. This complexity presents a challenge for investigating the molecular mechanisms between GWG and adverse pregnancy and birth outcomes using traditional approaches. With advances in omics technology, metabolome-wide association studies (MWASs) provide a systematic tool for the identification of novel metabolites and metabolic pathways related to health outcomes [8,9]. Such studies are extremely important for advancing our understanding of the underlying biological mechanisms that may explain the associations between GWG and health outcomes.

Recent MWASs have identified several metabolites/metabolic classes for weight gain in non-pregnant populations, with some metabolites being replicated across studies [10]. However, only one MWAS for GWG has been conducted among 37 Canadian women with the maternal metabolome measured at one time point between 25 and 28 weeks of gestation [11]. Given the unique and dynamic metabolic and physiological changes during pregnancy, the metabolome in pregnant women is distinct from non-pregnant women and is likely to change over the course of pregnancy [12,13]. To address the current research gaps, we performed an MWAS of GWG using untargeted plasma metabolomic profiling in a longitudinal study among pregnant women in which we modeled prospective, time-specific, and changes of metabolomic profiles during pregnancy in association with the total and excessive GWG. To gain a better understanding of the underlying pathways, we further examined the relationships of GWG-associated metabolites with three cardiometabolic biomarkers, including fasting plasma insulin, C-peptide, and high-sensitivity C-reactive protein (hs-CRP).

## 2. Materials and Methods

This study was ancillary to the Health in Pregnancy and Postpartum (HIPP) study, which was a randomized controlled trial conducted in South Carolina from 2015 to 2019 [14]. The original objective of the HIPP study was to investigate the efficacy of a behavioral lifestyle intervention on GWG. The study population included 219 pregnant women with prepregnancy overweight and obesity aged 18 to 44 years who self-identified as White or Black [14,15]. The ancillary study included 40 pregnant women from the HIPP study who agreed to provide their biospecimen for future research. All 40 women were recruited before 16 weeks of gestation (mean weeks at baseline = 12.1; standard deviation (SD) = 2.3). Participants included in the ancillary study and those not included had similar baseline demographics and lifestyles (Table S1). Among them, we excluded one woman without data on GWG, resulting in 39 women in the current analysis. All participants provided written informed consent. The current study was approved by the University of South Carolina Institutional Review Board.

The total GWG (in kg) was determined as the difference between the body weight at delivery on the medical chart and the self-reported prepregnancy weight at the screening visit. If the delivery room weight was unavailable, then the body weight at the last prenatal visit (average 4.8 days ahead of the delivery) was used to calculate the total GWG. A high correlation (r = 0.95) between the self-reported prepregnancy weight and the weight measured clinically was observed in our trial [15]. Excessive GWG was defined using the 2009 IOM criteria as a total GWG of over 11.5 kg for women with a prepregnancy BMI between 25 and 29.9 kg/m^2^ or a total GWG of over 9 kg for women with a prepregnancy BMI of 30 kg/m^2^ or above [2].

After the women consented to participate in the biospecimen ancillary study, trained staff collected fasting blood (EDTA tube) from the participants during their study visit at the Clinical Exercise Research Center (CERC). The samples were immediately processed and stored at −80 °C at the CERC until they were sent out for metabolomics analyses. All 39 women provided fasting blood samples in the first-trimester baseline visit (~12 weeks) and 29 of them also provided fasting blood samples in the third-trimester follow-up visit (~32 weeks). Thus, 39 fasting plasma samples were used for metabolomic profiling at 12 weeks, and 29 were used at 32 weeks. The fasting plasma concentrations of insulin, C-peptide, and hs-CRP at two time points (baseline and 32 weeks) were measured using the immunoturbidimetric and sandwich immunoassay methods, respectively, using the Roche COBAS 6000 chemistry analyzer (Roche Diagnostics, Indianapolis, IN, USA).

Untargeted metabolomic profiling of the fasting plasma samples at two time points (baseline and 32 weeks) was conducted by the West Coast Metabolomics Center, University of California Davis (UC-Davis) via combining three analytical platforms, including gas chromatography–time-of-flight mass spectrometry (GC-TOF MS, Leco, St. Joseph, MI, USA)), hydrophilic interaction chromatography–quadrupole time-of-flight tandem mass spectrometry (HILIC-QTOF MS/MS, Agilent, Santa Clara, CA), and charged-surface hybrid–quadrupole time-of-flight tandem mass spectrometry (CSH-QTOF MS/MS, Agilent, Santa Clara, CA, USA). Internal standards were employed for the identification of metabolites by matching the retention times. A total of 7514 features remained after blank filtering, including 769 structurally unique, annotated metabolites. Our calculations here focused on these 769 annotated metabolites with the classification of chemical compound classes performed using ClassyFire [16]. Provided the small percentage of missing values of the 769 annotated metabolites at baseline (0.3%) and 32 weeks (0.2%), we imputed missing values with half of the minimum value found in the dataset. Given the right-skewed distribution of metabolomics data, a natural log transformation was performed for all annotated metabolites [17]. To facilitate comparisons of the effect magnitudes across different metabolites, standardization (mean = 0, SD = 1) was also performed before performing the statistical analysis.

Maternal demographic variables, including maternal age, race/ethnicity, and parity were collected at the baseline visit at around 12 weeks using a study-specific questionnaire. The prepregnancy BMI was calculated based on the self-reported prepregnancy weight and height at the screening visit. Dietary intakes at baseline and 32 weeks were assessed using two automated self-administered 24-h dietary recalls (1 weekday and 1 weekend day) [18,19]. Physical activity (PA) at baseline and 32 weeks was assessed using the SenseWear Armband (CamNTech, Fenstanton, UK) for eight consecutive days. The SenseWear Armband is a multi-sensor accelerometer that was validated for assessing the PA of pregnant women [20]. The proprietary algorithms use the accelerometer and sensor data to classify the intensity of activity by metabolic equivalents. Only those who met the wear criteria (≥5 days, ≥1 weekend day, ≥21 h/day) were used to calculate the minutes/day spent in moderate-to-vigorous PA (MVPA). A total of 36 of 39 participants at baseline and 27 of 29 participants at 32 weeks met the wear criteria.

We compared the plasma metabolomic profiles between the intervention (*n* = 20) and control groups (*n* = 19) using principal component analysis and found no significant differences by intervention group (Figure S1). More importantly, the intervention and control groups achieved similar GWG at delivery in the HIPP study [15]. Thus, we combined the data from the two groups in the following analyses. First, we applied linear regression models to identify the metabolites at baseline that were individually associated with GWG, adjusting for age, race (White vs. Black), parity (0 vs. ≥1), and prepregnancy BMI. Then we performed least absolute shrinkage and selection operator (LASSO) regression models with 10-fold cross-validation, adjusted for the same covariates, to identify a panel of metabolites at baseline that were jointly associated with GWG [21]. Metabolites with non-zero coefficients based on the criteria of lambda.min were selected. For the metabolite panel selected in the LASSO regressions, we further estimated their predictability of excessive GWG using the area under (AUC) the receiver operating characteristic curve (ROC) in logistic regression models. The same procedures were repeated for metabolites at 32 weeks and percentage change in metabolites between baseline and 32 weeks (i.e., (metabolites_32weeks_ − metabolites_baseline_)/metabolites_baseline_). Moreover, we estimated the Pearson correlations between the metabolites individually associated with GWG and three cardiometabolic biomarkers measured at the same visit, including hs-CRP, C-peptide, and insulin. These biomarkers were log-transformed due to their right-skewed distribution. For linear regression analyses and Pearson correlation analyses, multiple comparisons were adjusted using the Benjamini–Hochberg correction, and a false discovery rate (FDR) of < 0.05 was considered statistically significant [22]. All data analyses were performed using R software (version 4.0.2. R Foundation for Statistical Computing, Vienna, Austria). 

## 3. Results

### 3.1. Demographic Characteristics

Among the 39 women, 38.5% were Black, 35.9% were nulliparous, 66.7% were married, 59.0% had a full-time job, and 30.8% were eligible for Medicaid. The mean maternal age was 30.4 (5.4) years, and the mean prepregnancy BMI was 33.3 (7.0) kg/m^2^, with 56.4% classified as obese (BMI ≥ 30 kg/m^2^). At baseline, the mean MVPA was 40.9 (23.0) minutes/day, and the mean total energy intake was 1869.1 (560.8) kcals/day (Table 1). The mean GWG was 12.4 (8.5) kg, with 27 (69.2%) females having excessive GWG. Additionally, there were no differences in the baseline characteristics, maternal pregnancy complications, or offspring conditions between the women with normal GWG and those with excessive GWG (Table S2).

### 3.2. Associations of Metabolites with GWG

Among the 769 annotated metabolites, there were 223 glycerophospholipids, 131 glycerolipids, 97 carboxylic acids and derivatives, 92 sphingolipids, 66 fatty acyls, 31 organooxygen compounds, 16 steroids and steroid derivatives, 13 indoles and derivatives, 13 organonitrogen compounds, 11 benzene and substituted derivatives, and 76 metabolites in classes that contained less than 10 metabolites (Figure S2).

**Figure 1:**
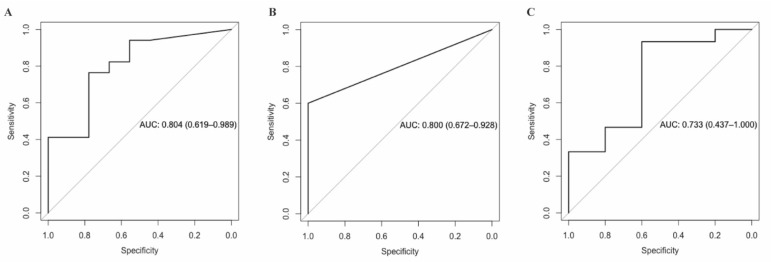
Receiver operating characteristic curves for panels of metabolites to predict excessive gestational weight gain. (**A**) Twelve metabolites at baseline around 12 weeks of gestation jointly associated with gestational weight gain (**B**) Four metabolites at 32 weeks of gestation jointly associated with gestational weight gain (**C**) Three changes of metabolites from baseline to 32 weeks of gestation jointly associated with gestational weight gain. Participants were randomly divided into training and validation sets with a ratio of 1:2, based on which the area under the curve (AUC) was calculated using logistic regression models.

At baseline, a total of 104 metabolites were associated with GWG with raw *p*-values < 0.05, but none of them were significant after FDR correction (all FDR > 0.05) (Table S3). A panel of 12 metabolites was jointly associated with the total GWG in LASSO regression with 10-fold cross-validation (Table 2). The AUC for excessive GWG of this 12-metabolite panel was 0.80 (95% CI: 0.62, 0.99; *p* = 0.01) (Figure 1A).

**Figure 2:**
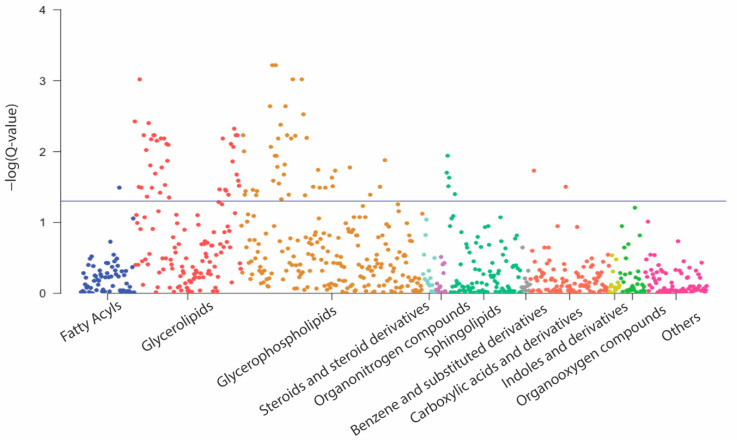
Manhattan plot for associations between metabolites at 32 weeks of gestation and total gestational weight gain (GWG) and by metabolic class in linear regression models adjusted for age, race/ethnicity, parity, and prepregnancy BMI and corrected for the multiple comparisons using the Benjamini–Hochberg procedure, with false discovery rates (FDRs) of < 0.05 as the statistically significant level. The horizontal lines represented the adjusted *p* values (i.e., Q value) of 0.05. “Others” included 76 metabolites in classes that contained less than 10 metabolites.

At 32 weeks, 186 metabolites were associated with GWG with raw *p*-values of < 0.05 (Table S4), while 88 were significantly and positively associated with GWG after adjusting for multiple comparisons (FDR < 0.05), including 42 glycerophospholipids (i.e., 35 phosphatidylcholines (PC) and 7 lysophosphatidylcholines (LPC)), 38 triglycerides (TAG), 5 sphingomyelins (SM), and one polyunsaturated fatty acid (i.e., arachidonic acid), while only two were amino acid-related (i.e., isoleucine, and Phe-Trp) (Figure 2). A panel of four metabolites at 32 weeks was jointly associated with GWG in LASSO regression with 10-fold cross-validation, including PC (34:4), TAG (52:6), arachidonic acid, and isoleucine (Table 2). The AUC for excessive GWG of this four-metabolite panel was 0.80 (95% CI: 0.67, 0.93; *p* = 0.02) (Figure 1B). All of them were also individually associated with total GWG (FDR < 0.05).

For changes in the metabolites from baseline to 32 weeks, 47 metabolites were associated with GWG (raw *p*-values < 0.05), but none of them were significant after FDR correction (FDR > 0.05) (Table S5). A panel of three metabolite changes (i.e., from baseline to 32 weeks) was jointly associated with GWG in LASSO regression with 10-fold cross-validation (Table 2). The AUC for excessive GWG of this four-metabolite panel was 0.73 (95% CI: 0.44, 1.00; *p* = 0.07) (Figure 1C).

### 3.3. Correlations between Metabolites and Cardiometabolic Biomarkers

Of the 88 metabolites individually associated with GWG around 32 weeks, seven TAGs (i.e., TAG (52:4), TAG (52:6; 14:0-18:2-20:4), TAG (48:3), TAG (48:5), TAG (50:5), TAG (54:5)B, TAG (54:6)B) were nominally correlated (*p* < 0.05) with both insulin and C-peptide collected at 32 weeks. However, no correlations were significant after FDR correction (FDR > 0.05) (Table 3).

## 4. Discussion

In this longitudinal study among Black and White pregnant women with overweight and obesity and untargeted metabolomic profiling, we identified novel blood metabolites associated with total GWG. In the third trimester (~32 weeks), 88 metabolites were individually associated with GWG in linear regressions, independent of age, race/ethnicity, parity, and prepregnancy BMI. A panel of four metabolites (i.e., PC (34:4), TAG (52:6), isoleucine, and arachidonic acid) was jointly associated with GWG in LASSO regressions, which was also consistently identified in linear regression models. These four metabolites well predicted excessive GWG with an AUC of 0.80. No significant correlation was observed for the 88 metabolites that were individually associated with GWG and three cardiometabolic biomarkers (i.e., insulin, C-peptide, and hs-CRP) at 32 weeks. We also identified a panel of 12 metabolites at baseline with an AUC of 0.80 and a panel of three changes of metabolites from baseline to 32 weeks with an AUC of 0.73, which well predicted excessive GWG, though they were not individually associated with the total GWG.

Two studies have investigated GWG-related metabolites using a targeted approach (i.e., the measurement of defined groups of chemically characterized and biochemically annotated metabolites) [23] or an untargeted approach but focusing solely on lipid metabolites (i.e., lipidomics) [24]. Hellmuth et al. performed targeted profiling on amino acids and non-esterified fatty acids (NEFA) and examined cross-sectional associations in 160 U.S. pregnant women (93 women with a normal prepregnancy BMI) [23]. Two metabolites, α-ketoglutaric acid in the first and third trimesters and SM.a.C30.1 in the first trimester, were associated with the trimester-specific GWG [23]. Lau et al., conducted lipidomic profiling using non-fasting plasma samples collected from 114 U.S. pregnant women with prepregnancy overweight or obesity at 15 and 35 weeks and found the changes of 17 lipids, mainly PCs and SMs, were associated with GWG [24]. To date, only one study, recently conducted among 37 Canadian pregnant women by Shearer et al., performed untargeted metabolomic profiling and an MWAS of GWG. Four metabolites (i.e., glutamate, lysine, pyruvate, and valine) in the second trimester (25–28 weeks) were individually associated with excessive GWG in this study [11]. In contrast to our study, the Shearer et al. study used nuclear magnetic resonance (NMR) spectroscopy for the untargeted metabolomic profiling and non-fasting blood samples and included mainly lean women (23 women (65%) with normal prepregnancy BMI) [11] who were at lower risk of excessive GWG [3]. No metabolites have been replicated across studies (including the current study), which might be explained by the different platforms for metabolomic profiling (i.e., NMR vs. GC/MS) [25], different biospecimens (i.e., fasting vs. non-fasting), and the heterogeneity of the study populations. It is noteworthy that three of the four metabolites at 32 weeks (i.e., isoleucine, arachidonic acid [26,27], and PC (34:4) [28,29]) that were both individually and jointly associated with the total GWG and excessive GWG during pregnancy in our study were also associated with obesity in previous studies in non-pregnant populations.

A large body of evidence has suggested that obesity is accompanied by an increased level of circulating branched-chain amino acids (BCAAs) [30]. Isoleucine, a BCAA, was positively associated with GWG in our study and in previous studies among non-pregnant adults and children [30]. Interestingly, another BCAA, valine, was associated with GWG in the Shearer et al. study [11]. Collectively, the available data tend to indicate the importance of BCAAs and their metabolic pathways in the etiology of obesity in both non-pregnant populations and pregnant women.

Our study observed a significant and positive association between arachidonic acid and GWG in pregnant women, which is consistent with previous studies conducted in children and adults with obesity in Europe [26,27]. As an omega-6 polyunsaturated fatty acid (PUFA), the biological functions of arachidonic acid have been studied intensively, including the initiation and resolution of inflammation, the adaption of mood and appetite, and the pathophysiology of obesity [31]. Specifically, 5-, 11-, and 15-hydroxyeicosatetraenoic acids, the derivatives of arachidonic acid, were also positively associated with BMI, waist circumference, and serum leptin, indicating a potential pathway between arachidonic acid and GWG [32].

Phosphatidylcholines (PCs) are major components of eukaryotic cell membranes and are engaged in membrane trafficking and inflammation [33]. The relationships of each subtype of PCs with health outcomes are not well understood but are likely varied by the length of the carbon chains and the number and location of the double bonds [34]. A previous study found that a higher level of plasma PC (34:4) was associated with an increased risk of obesity in adults, with the association being independent of genetic factors in a small twin study [28]. In addition, a recent study observed a decline of the plasma PC (34:4) concentration after an eight-week weight loss intervention (an average weight loss of 9.7 kg and total fat reduction of 7.2 kg) among 162 adults, suggesting that PC (34:4) may play a role in obesity development and adiposity accumulation [29].

Increased plasma levels of the total TAGs are one of the characteristics of dyslipidemia commonly occurring in obesity [35]. Long-chain fatty acids are the most common type of TAGs and are mainly used as energy sources in humans [36]. Their specific function, however, may vary by the length of carbon chains and the number and location of double bonds. For instance, TAG (48:0), TAG (48:1), and TAG (50:5) have been reported for their predictability of the future development of type 2 diabetes among individuals in the Netherlands [37]. TAG (16:0/18:1/22:5), TAG (18:1/18:1/20:4), and TAG (16:0/18:1/18:1) have been associated with calcium scores among patients with calcific coronary artery disease [38]. However, to our best knowledge, no studies have reported the association between specific long-chain TAGs and obesity.

Our study has several unique strengths, including the prospective design, a large proportion (38.5%) of Black women, untargeted metabolomic profiling using three platforms, and the availability of fasting blood samples collected at two time points across the pregnancy. Given the dynamic changes of blood metabolites after meals, fasting blood samples are less likely to be influenced by the last meals and are more likely preferred in metabolomics studies [39]. There are also several limitations to be acknowledged. First, we had a modest sample size, which may limit the study’s power to identify some GWG-related metabolites. However, most metabolomics studies have had similar sample sizes given the expenses associated with untargeted metabolomic profiling. The research field has recognized this limitation and called for establishing metabolomics consortiums (e.g., COMETS) to aggregate datasets and resources [25]. Second, although we collected fasting plasma samples in both the first and the third trimesters, no fasting plasma sample was collected in the second trimester [40]. Future studies collecting fasting blood samples in the second trimester may provide a novel perspective on the metabolic biomarkers of GWG. Third, our study included only pregnant women with overweight and obesity, which may limit the generalization of the study findings to those with under or normal prepregnancy weight. Given that about 56% of U.S women were overweight (26.7%) or obese (29.5%) in 2020 [41] and our inclusion of black and white women, the study findings could still be appropriately applied to the majority of U.S. pregnant women. Nevertheless, results from our study need to be replicated by other studies, particularly with a larger sample size. In addition, future studies can explore the medication effect of prepregnancy BMI (e.g., normal weight vs. overweight and obesity) on the associations between metabolites and GWG. Finally, as an observational study, this study may inevitably suffer from residual confounding (e.g., genetic factors), though several key confounders have been controlled.

In conclusion, our study identified some novel metabolomic markers of GWG in both the first and the third trimesters, with some of them potentially related to the pathophysiology of GWG and others as promising predictors for excessive GWG. Future studies with a larger sample size are warranted to replicate our findings and explore the molecular mechanisms of the potential pathophysiology of GWG.

## Figures and Tables

**Table 1:** Maternal demographic characteristics for the 39 pregnant women at baseline (around 12 weeks of gestation).

Characteristics ^1^	*n* = 39
**Demographic characteristics**	
Gestational age at baseline (week, mean (SD))	12.09 (2.26)
Age (year, mean (SD))	30.41 (5.41)
Prepregnancy BMI (kg/m^2^, mean (SD))	33.25 (7.04)
Obese (%)	22 (56.4)
Nulliparous (%)	14 (35.9)
Black (%)	15 (38.5)
Married (%)	26 (66.7)
Full time employed (%)	23 (59.0)
Medicaid use (%)	12 (30.8)
**Physical activity at baseline (mean (SD)) ^2^**	
Moderate physical activity (min/day)	40.35 (21.99)
Vigorous physical activity (min/day)	0.33 (0.87)
Moderate to vigorous physical activity (min/day)	40.88 (23.00)
Steps per day	5727.62 (2427.90)
**Dietary intake at baseline (mean (SD))**	
Total energy (kcal/day)	1869.05 (560.79)
Total protein (g/day)	73.04 (22.46)
Total fatty acids (g/day)	75.31 (26.25)
Saturated fatty acids (g/day)	24.53 (10.60)
Monounsaturated fatty acids (g/day)	26.33 (8.98)
Polyunsaturated fatty acids (g/day)	18.41 (7.95)
Cholesterol (mg/day)	269.94 (150.32)
Total HEI—2015 score ^3^	52.98 (14.31)
**Maternal pregnancy complications**	
Gestational diabetes (%)	3 (7.7)
Gestational hypertension (%)	8 (20.5)
**Offspring conditions**	
Girl (%)	19 (48)
Low birth weight (%) ^4^	2 (5.1)
Preterm birth (%)^4^	1 (2.6)
Small for gestational age (%) ^4^	4 (10.3)
Large for gestational age (%) ^4^	4 (10.3)

^1^ Data are presented as frequency (percentage) for categorical variables and mean (standard deviations, SD) for continuous variables. ^2^ Three pregnant women had missing data on physical activity. ^3^ The HEI–2015 scores ranged from 0 to 100, with a higher HEI score reflecting better adherence to the 2015–2020 Dietary Guidelines for Americans. ^4^ One offspring had missing data on low birth weight, preterm birth, small for gestational age, and large for gestational age. Abbreviations: BMI, body mass index; HEI, healthy eating index; SD, standard deviation.

**Table 2:** Metabolite panels associated with total gestational weight gain in LASSO regression ^1^.

12 Weeks of Gestation			32 Weeks of Gestation			Changes from 12 to 32 Weeks of Gestation		
Metabolite	Class	Direction	Metabolite	Class	Direction	Metabolite	Class	Direction
PC (40:6) A	Glycerophospholipids	Positive	PC (34:4)	Glycerophospholipids	Positive	PC (40:7) A	Phosphatidylcholine	Negative
TAG (43:0) or TAG (13:0-14:0-16:0)	Glycerolipids	Positive	TAG (52:6)	Glycerolipids	Positive	TAG (60:4)	Glycerolipids	Negative
SM (d30:1)	Sphingolipids	Positive	Arachidonic acid	Omega-6 PUFA	Positive	Adenosine	Purine nucleosides	Positive
SM (d32:0)	Sphingolipids	Positive	Isoleucine	Amino acid	Positive			
PE (p-38:2) or PE (o-38:3)	Glycerophospholipids	Positive						
3-Hydroxybutyrylcarnitine	Fatty Acyls	Negative						
Methyltestosterone	Steroids and steroid derivatives	Positive						
Aconitic acid	Carboxylic acids and derivatives	Negative						
1-methylgalactose	Organooxygen compounds	Positive						
Trans-3’-Hydroxycotinine	Pyridines and derivatives	Positive						
Trigonelline	Alkaloids	Negative						
Adipic acid	Carboxylic acids and derivatives	Positive						

^1^ LASSO regression adjusted for maternal age, race, parity, and prepregnancy BMI with 10-fold cross-validation (lambda.min). Abbreviations: BMI, body mass index; LASSO, least absolute shrinkage and selection operator; PC, phosphatidylcholine; PE, phosphatidylethanolamines; SM, sphingomyelin; TAG, triacylglycerol, PUFA: polyunsaturated fatty acids.

**Table 3:** Pearson correlations between 88 metabolites and three cardiometabolic biomarkers at 32 weeks of gestation.

			Insulin			C-Peptide			Hs-CRP		
Metabolite ^1^	Superclass	Class	β	P	FDR	β	P	FDR	β	P	FDR
Arachidonic acid	Lipids and lipid-like molecules	Fatty Acyls	0.14	0.46	0.70	−0.08	0.68	0.84	−0.12	0.52	0.88
TAG 45:0 or TAG 14:0-15:0-16:0	Lipids and lipid-like molecules	Glycerolipids	0.15	0.44	0.68	0.22	0.26	0.50	−0.12	0.54	0.88
TAG 45:1 or TAG 12:0-16:0-17:1	Lipids and lipid-like molecules	Glycerolipids	0.15	0.43	0.68	0.21	0.27	0.50	−0.11	0.57	0.88
TAG 46:2 or TAG 12:0-16:1-18:1	Lipids and lipid-like molecules	Glycerolipids	0.19	0.33	0.68	0.21	0.28	0.50	−0.30	0.11	0.88
TAG 47:1 or TAG 15:0-16:0-16:1	Lipids and lipid-like molecules	Glycerolipids	0.10	0.59	0.78	0.23	0.24	0.49	0.00	0.99	0.99
TAG 47:2 or TAG 14:0-15:0-18:2	Lipids and lipid-like molecules	Glycerolipids	0.13	0.51	0.71	0.24	0.22	0.47	−0.13	0.50	0.88
TAG 48:3 or TAG 14:0-16:1-18:2	Lipids and lipid-like molecules	Glycerolipids	0.17	0.39	0.68	0.23	0.22	0.47	−0.29	0.13	0.88
TAG 49:3 or TAG 15:0-16:1-18:2	Lipids and lipid-like molecules	Glycerolipids	0.09	0.65	0.82	0.26	0.17	0.45	−0.02	0.91	0.94
TAG 50:5 or TAG 14:1-18:2-18:2	Lipids and lipid-like molecules	Glycerolipids	0.30	0.11	0.68	0.36	0.06	0.31	−0.35	0.06	0.88
TAG 52:4	Lipids and lipid-like molecules	Glycerolipids	0.41	0.03	0.37	0.45	0.01	0.31	−0.07	0.70	0.88
TAG 52:6 or TAG 14:0-18:2-20:4	Lipids and lipid-like molecules	Glycerolipids	0.38	0.04	0.37	0.38	0.04	0.31	−0.23	0.23	0.88
TAG 58:7 or TAG 18:0-18:2-22:5	Lipids and lipid-like molecules	Glycerolipids	0.38	0.04	0.37	0.37	0.05	0.31	−0.21	0.28	0.88
TAG (40:1)	Lipids and lipid-like molecules	Glycerolipids	0.28	0.14	0.68	0.30	0.12	0.39	−0.08	0.69	0.88
TAG (44:0)	Lipids and lipid-like molecules	Glycerolipids	0.19	0.32	0.68	0.22	0.26	0.50	−0.21	0.27	0.88
TAG (44:1)	Lipids and lipid-like molecules	Glycerolipids	0.13	0.51	0.71	0.19	0.33	0.57	−0.18	0.34	0.88
TAG (44:2)	Lipids and lipid-like molecules	Glycerolipids	0.22	0.26	0.68	0.17	0.38	0.61	−0.33	0.08	0.88
TAG (46:0)	Lipids and lipid-like molecules	Glycerolipids	0.17	0.39	0.68	0.21	0.28	0.50	−0.19	0.33	0.88
TAG (46:1)	Lipids and lipid-like molecules	Glycerolipids	0.16	0.40	0.68	0.22	0.25	0.50	−0.17	0.38	0.88
TAG (46:3) A	Lipids and lipid-like molecules	Glycerolipids	0.22	0.25	0.68	0.26	0.17	0.45	−0.30	0.11	0.88
TAG (48:1)	Lipids and lipid-like molecules	Glycerolipids	0.21	0.27	0.68	0.31	0.11	0.39	−0.09	0.65	0.88
TAG (48:2)	Lipids and lipid-like molecules	Glycerolipids	0.24	0.22	0.68	0.31	0.10	0.39	−0.17	0.38	0.88
TAG (48:3)	Lipids and lipid-like molecules	Glycerolipids	0.42	0.02	0.37	0.40	0.03	0.31	−0.18	0.34	0.88
TAG (48:4) B	Lipids and lipid-like molecules	Glycerolipids	0.37	0.05	0.40	0.36	0.06	0.31	−0.26	0.18	0.88
TAG (48:5)	Lipids and lipid-like molecules	Glycerolipids	0.41	0.03	0.37	0.41	0.03	0.31	−0.18	0.34	0.88
TAG (49:2)	Lipids and lipid-like molecules	Glycerolipids	0.08	0.67	0.83	0.25	0.18	0.45	0.08	0.68	0.88
TAG (49:3)	Lipids and lipid-like molecules	Glycerolipids	0.11	0.58	0.78	0.27	0.16	0.45	0.03	0.89	0.94
TAG (50:2)	Lipids and lipid-like molecules	Glycerolipids	0.26	0.16	0.68	0.40	0.03	0.31	−0.02	0.90	0.94
TAG (50:3) A	Lipids and lipid-like molecules	Glycerolipids	0.22	0.25	0.68	0.37	0.05	0.31	−0.10	0.59	0.88
TAG (50:5)	Lipids and lipid-like molecules	Glycerolipids	0.39	0.03	0.37	0.37	0.05	0.31	−0.19	0.31	0.88
TAG (52:5)	Lipids and lipid-like molecules	Glycerolipids	0.16	0.42	0.68	0.24	0.20	0.46	−0.05	0.80	0.89
TAG (52:6)	Lipids and lipid-like molecules	Glycerolipids	0.19	0.32	0.68	0.30	0.11	0.39	−0.17	0.37	0.88
TAG (53:5)	Lipids and lipid-like molecules	Glycerolipids	0.16	0.40	0.68	0.30	0.12	0.39	0.07	0.72	0.88
TAG (54:5) B	Lipids and lipid-like molecules	Glycerolipids	0.40	0.03	0.37	0.47	0.01	0.31	−0.07	0.74	0.88
TAG (54:6) B	Lipids and lipid-like molecules	Glycerolipids	0.47	0.01	0.37	0.45	0.01	0.31	−0.21	0.28	0.88
TAG (54:7) A	Lipids and lipid-like molecules	Glycerolipids	0.40	0.03	0.37	0.36	0.05	0.31	−0.32	0.09	0.88
TAG (54:7) B	Lipids and lipid-like molecules	Glycerolipids	0.33	0.08	0.53	0.37	0.05	0.31	−0.18	0.34	0.88
TAG (54:8)	Lipids and lipid-like molecules	Glycerolipids	0.18	0.36	0.68	0.30	0.11	0.39	−0.28	0.15	0.88
TAG (56:9)	Lipids and lipid-like molecules	Glycerolipids	0.21	0.27	0.68	0.34	0.07	0.35	−0.19	0.32	0.88
TAG (58:9)	Lipids and lipid-like molecules	Glycerolipids	0.22	0.26	0.68	0.20	0.30	0.53	−0.08	0.67	0.88
LPC (14:0)	Lipids and lipid-like molecules	Glycerophospholipids	0.02	0.90	0.94	0.16	0.40	0.62	−0.15	0.45	0.88
LPC (16:1)	Lipids and lipid-like molecules	Glycerophospholipids	0.09	0.65	0.82	0.27	0.16	0.45	0.11	0.57	0.88
LPC (20:3)	Lipids and lipid-like molecules	Glycerophospholipids	0.17	0.37	0.68	0.28	0.14	0.44	0.14	0.47	0.88
LPC (14:0)	Lipids and lipid-like molecules	Glycerophospholipids	0.15	0.43	0.68	0.27	0.16	0.45	−0.25	0.19	0.88
LPC (16:1)	Lipids and lipid-like molecules	Glycerophospholipids	0.19	0.32	0.68	0.36	0.06	0.31	0.06	0.76	0.88
LPC (20:3)	Lipids and lipid-like molecules	Glycerophospholipids	0.25	0.20	0.68	0.35	0.06	0.31	0.06	0.76	0.88
LPC (22:5)	Lipids and lipid-like molecules	Glycerophospholipids	0.01	0.97	0.97	0.07	0.72	0.84	0.28	0.14	0.88
PC (32:1)	Lipids and lipid-like molecules	Glycerophospholipids	0.03	0.86	0.91	0.14	0.48	0.72	0.07	0.71	0.88
PC (32:2)	Lipids and lipid-like molecules	Glycerophospholipids	−0.04	0.83	0.91	0.02	0.93	0.95	−0.27	0.15	0.88
PC (33:1)	Lipids and lipid-like molecules	Glycerophospholipids	−0.28	0.15	0.68	−0.07	0.71	0.84	0.16	0.40	0.88
PC (34:3)	Lipids and lipid-like molecules	Glycerophospholipids	0.15	0.43	0.68	0.23	0.22	0.47	−0.04	0.82	0.89
PC (34:4)	Lipids and lipid-like molecules	Glycerophospholipids	−0.01	0.97	0.97	0.07	0.70	0.84	−0.17	0.37	0.88
PC (36:3) A	Lipids and lipid-like molecules	Glycerophospholipids	0.10	0.61	0.79	0.26	0.17	0.45	0.05	0.82	0.89
PC (36:5) B	Lipids and lipid-like molecules	Glycerophospholipids	−0.20	0.30	0.68	−0.04	0.83	0.91	0.12	0.53	0.88
PC (40:6) A	Lipids and lipid-like molecules	Glycerophospholipids	0.01	0.96	0.97	−0.02	0.91	0.95	−0.04	0.83	0.89
PC (28:0)	Lipids and lipid-like molecules	Glycerophospholipids	−0.05	0.79	0.91	0.02	0.93	0.95	−0.19	0.33	0.88
PC (30:0)	Lipids and lipid-like molecules	Glycerophospholipids	0.04	0.84	0.91	0.11	0.59	0.77	−0.10	0.61	0.88
PC (31:0)	Lipids and lipid-like molecules	Glycerophospholipids	−0.22	0.26	0.68	−0.04	0.85	0.91	0.15	0.44	0.88
PC (31:1)	Lipids and lipid-like molecules	Glycerophospholipids	−0.11	0.58	0.78	0.06	0.77	0.88	0.14	0.46	0.88
PC (32:1)	Lipids and lipid-like molecules	Glycerophospholipids	0.05	0.78	0.91	0.17	0.37	0.60	0.09	0.64	0.88
PC (32:2)	Lipids and lipid-like molecules	Glycerophospholipids	−0.04	0.82	0.91	0.04	0.84	0.91	−0.25	0.19	0.88
PC (33:0)	Lipids and lipid-like molecules	Glycerophospholipids	−0.19	0.31	0.68	−0.01	0.97	0.98	0.18	0.36	0.88
PC (33:1)	Lipids and lipid-like molecules	Glycerophospholipids	−0.14	0.47	0.70	0.06	0.75	0.87	0.19	0.33	0.88
PC (34:3) A	Lipids and lipid-like molecules	Glycerophospholipids	−0.11	0.57	0.78	0.09	0.65	0.82	0.10	0.59	0.88
PC (34:3) B	Lipids and lipid-like molecules	Glycerophospholipids	0.20	0.29	0.68	0.25	0.18	0.45	−0.11	0.56	0.88
PC (34:3) C	Lipids and lipid-like molecules	Glycerophospholipids	0.23	0.23	0.68	0.32	0.09	0.39	−0.01	0.95	0.97
PC (34:4)	Lipids and lipid-like molecules	Glycerophospholipids	0.04	0.85	0.91	0.12	0.53	0.74	−0.17	0.38	0.88
PC (35:4)	Lipids and lipid-like molecules	Glycerophospholipids	−0.34	0.08	0.53	−0.10	0.59	0.77	0.11	0.58	0.88
PC (36:3) B	Lipids and lipid-like molecules	Glycerophospholipids	0.16	0.40	0.68	0.34	0.08	0.35	0.08	0.69	0.88
PC (36:4) B	Lipids and lipid-like molecules	Glycerophospholipids	0.16	0.41	0.68	0.24	0.20	0.46	−0.05	0.78	0.88
PC (36:5) C	Lipids and lipid-like molecules	Glycerophospholipids	−0.05	0.81	0.91	0.13	0.51	0.73	0.14	0.46	0.88
PC (36:5) D	Lipids and lipid-like molecules	Glycerophospholipids	−0.16	0.40	0.68	0.00	0.98	0.98	0.10	0.62	0.88
PC (36:6)	Lipids and lipid-like molecules	Glycerophospholipids	−0.08	0.68	0.83	0.09	0.64	0.81	−0.14	0.46	0.88
PC (37:5)	Lipids and lipid-like molecules	Glycerophospholipids	−0.38	0.04	0.37	−0.14	0.46	0.70	0.08	0.70	0.88
PC (38:3)	Lipids and lipid-like molecules	Glycerophospholipids	0.25	0.19	0.68	0.30	0.12	0.39	0.12	0.53	0.88
PC (38:4) B	Lipids and lipid-like molecules	Glycerophospholipids	0.26	0.18	0.68	0.25	0.20	0.46	0.05	0.78	0.88
PC (38:6) A	Lipids and lipid-like molecules	Glycerophospholipids	−0.05	0.80	0.91	−0.07	0.71	0.84	−0.08	0.67	0.88
PC (38:6) C	Lipids and lipid-like molecules	Glycerophospholipids	0.18	0.34	0.68	0.38	0.04	0.31	0.09	0.63	0.88
PC (38:7)	Lipids and lipid-like molecules	Glycerophospholipids	−0.13	0.49	0.71	0.11	0.57	0.77	0.15	0.44	0.88
PC (40:7) A	Lipids and lipid-like molecules	Glycerophospholipids	0.06	0.76	0.91	0.14	0.47	0.72	0.12	0.55	0.88
PC (40:8)	Lipids and lipid-like molecules	Glycerophospholipids	−0.01	0.95	0.97	−0.02	0.93	0.95	0.07	0.73	0.88
PC (42:6)	Lipids and lipid-like molecules	Glycerophospholipids	0.17	0.37	0.68	0.21	0.28	0.50	0.08	0.69	0.88
SM (d30:1) A	Lipids and lipid-like molecules	Sphingolipids	−0.19	0.32	0.68	−0.13	0.50	0.73	0.08	0.68	0.88
SM (d32:2) A	Lipids and lipid-like molecules	Sphingolipids	−0.13	0.50	0.71	0.04	0.83	0.91	0.08	0.69	0.88
SM (d30:1) B	Lipids and lipid-like molecules	Sphingolipids	−0.18	0.36	0.68	−0.10	0.60	0.77	0.07	0.72	0.88
SM (d32:0)	Lipids and lipid-like molecules	Sphingolipids	−0.24	0.20	0.68	−0.11	0.59	0.77	0.13	0.50	0.88
SM (d32:2) B	Lipids and lipid-like molecules	Sphingolipids	−0.04	0.83	0.91	0.12	0.53	0.74	0.07	0.73	0.88
Isoleucine	Organic acids and derivatives	Carboxylic acids and derivatives	0.25	0.19	0.68	0.05	0.79	0.89	0.01	0.97	0.98
Phe-Trp	Organic acids and derivatives	Carboxylic acids and derivatives	0.24	0.20	0.68	0.18	0.36	0.60	0.09	0.64	0.88

^1^ Metabolites at 32 weeks that were individually associated with total GWG in multiple linear regression models adjusted for maternal age, race, parity, and prepregnancy BMI (FDR < 0.05). Abbreviations: BMI, body mass index; FDR, false-discovery rate; GWG, gestational weight gain; hs-CRP: high-sensitive C-reactive protein; LPC, lysophosphatidylcolines; PC, phosphatidylcholine; PE, phosphatidylethanolamine; SM, sphingomyelin; TAG, triacylglycerol.

## Data Availability

The datasets generated during and/or analyzed during the current study are available from the corresponding author upon reasonable request. The data are not publicly available due to the fact that study participants did not agree for their data to be shared publicly.

## Supplementary Materials

The following supporting information can be downloaded at: https://www.mdpi.com/article/10.3390/metabo12100960/s1, Table S1: Comparison of baseline characteristics among patients included in the ancillary study and those only included in the HIPP study; Table S2: Comparison of baseline characteristics, maternal pregnancy complications, and offspring conditions between participants with normal gestational weight gain versus those with excessive gestational weight gain in the current study; Table S3: Associations of 104 metabolites at baseline around 12 weeks of gestation with gestational weight gain in linear regression analyses; Table S4: Associations of 186 metabolites at 32 weeks of gestation with gestational weight gain in linear regression analyses; Table S5: Associations of 47 changes of metabolites from baseline to 32 weeks of gestation with gestational weight gain in linear regression analyses; Figure S1: Principal component analysis score plot of the first two principal components of 29 pregnant women based on their metabolites collected around 32 weeks of gestation; Figure S2: Dendrogram of the analyzed metabolites by superclass and class, for which the classification of chemical compound was performed using ClassyFire. STROBE Statement for cohort studies was included.
