# Supplementary material for: Metabolome-Wide Associations of Gestational Weight Gain in Pregnant Women with Overweight and Obesity
Source: Metabolites. 2022 Oct 11;12(10):960. doi: 10.3390/metabo12100960 (PMC9609233; doi:10.3390/metabo12100960)
Supplement: Supplementary file 1 [file metabolites-12-00960-s001.zip › metabolites-1925435-supplementary.pdf]

Supplementary Materials

Metabolome-Wide Associations of Gestational Weight Gain in Pregnant Women with Overweight and Obesity

Jin Dai [1], Nansi S. Boghossian [2], Mark A. Sarzynski [3], Feng Luo [4], Xiaoqian Sun [5], Jian Li [6,7], Oliver Fiehn [8], Jihong Liu [2,*] and Liwei Chen [1,*]

[1] Department of Epidemiology, Fielding School of Public Health, University of California, Los Angeles, CA 90095, USA
[2] Department of Epidemiology and Biostatistics, Arnold School of Public Health, University of South Carolina, Columbia, SC 29208, USA
[3] Department of Exercise Science, Arnold School of Public Health, University of South Carolina, Columbia, SC 29208, USA
[4] School of Computing, Clemson University, Clemson, SC 29634, USA
[5] Department of Mathematical and Statistical Sciences, Clemson University, Clemson, SC 29634, USA
[6] Department of Environmental Health Sciences, Fielding School of Public Health, University of California, Los Angeles, CA 90095, USA
[7] School of Nursing, University of California, Los Angeles, CA 90095, USA
[8] West Coast Metabolomics Center, University of California, Davis, CA 95616, USA
* Correspondence: jliu@mailbox.sc.edu (J.L.); cliwei86@ucla.edu (L.C.)

Table S1. Comparison of baseline characteristics between patients included in the ancillary study and those only included in the HIPP study.

	**Yes**	**No**	**P-values[2]**
**Characteristics[1]**	***n*** **= 39**	***n*** **= 179**	
**Demographic characteristics**			
Gestational age at baseline (week, mean (SD))	12.09 (2.26)	12.72 (2.32)	0.10
Age (year, mean (SD))	30.41 (5.41)	29.58 (4.96)	0.31
Prepregnancy BMI (kg/m2, mean (SD))	33.25 (7.04)	33.40 (6.17)	0.84
Obese (%)	22 (56.4)	90 (50.3)	0.52
Multiparous (%)	14 (35.9)	99 (55.3)	0.35
Black (%)	15 (38.5)	82 (45.8)	0.44
Married (%)	26 (66.7)	121 (67.6)	0.90
Full time employed (%)	23 (59.0)	110 (61.5)	1
Medicaid use (%)	12 (30.8)	55 (30.7)	1
**Physical activity at Baseline (mean (SD))**			
Moderate physical activity (min/day)	40.35 (21.99)	35.51 (21.97)	0.28
Vigorous physical activity (min/day)	0.33 (0.87)	0.31 (1.07)	0.97
Moderate to vigorous physical activity (min/day)	40.88 (23.00)	35.84 (22.22)	0.27
Steps per day	5727.62 (2427.90)	5288.25 (2107.20)	0.31
**Dietary Intake at Baseline (mean (SD))**			
Total Energy (kcal/day)	1869.05 (560.79)	1946.77 (636.86)	0.50
Total Protein (g/day)	73.04 (22.46)	80.87 (31.65)	0.16
Total Fatty acids (g/day)	75.31 (26.25)	80.18 (30.16)	0.33
Saturated fatty acids (g/day)	24.53 (10.60)	26.81 (11.57)	0.26
Monounsaturated fatty acids (g/day)	26.33 (8.98)	28.26 (11.41)	0.29
Polyunsaturated fatty acids (g/day)	18.41 (7.95)	18.38 (7.97)	0.91
Cholesterol (mg/day)	269.94 (150.32)	306.13 (174.76)	0.21
Total HEI-2015 score[3]	52.98 (14.31)	51.89 (11.23)	0.71

[1] Data are presented as frequency (percentage) for categorical variables and mean (standard deviations, SD) for continuous variables.
[2] P-values were calculated using t-tests for continuous variables and χ^2-tests for categorical variables.
[3] The HEI-2015 scores range from 0 to 100, with a higher HEI score reflecting better adherence to the 2015-2020 Dietary Guidelines for Americans.
Abbreviations: BMI, body mass index; HEI, healthy eating index; SD, standard deviation.

Table S2. Comparison of baseline characteristics, maternal pregnancy complications, and offspring conditions between participants with normal gestational weight gain versus those with excessive gestational weight gain in the current study.

Characteristics[1]	Normal GWG *n* = 12	Excessive GWG[2] *n* = 27	P-value[3]
**Demographic characteristics**			
Gestational age at baseline (week, mean (SD))	11.42 (2.86)	12.39 (1.93)	0.22
Age (year, mean (SD))	29.58 (5.35)	30.78 (5.49)	0.53
Prepregnancy BMI (kg/m2, mean (SD))	32.38 (5.24)	33.64 (7.77)	0.61
Obese (%)	8 (66.7)	4 (51.9)	0.61
Nulliparous (%)	7 (58.3)	8 (66.7)	0.89
Black (%)	4 (33.3)	11 (40.7)	0.93
Married (%)	7 (58.3)	19 (70.4)	0.71
Full-time employed (%)	8 (66.7)	15 (55.6)	0.77
Medicaid use (%)	6 (50.0)	6 (22.2)	0.17
**Physical activity at Baseline (mean (SD))**			
Moderate physical activity (min/day)	49.26 (25.36)	36.43 (19.61)	0.11
Vigorous physical activity (min/day)	0.71 (1.46)	0.16 (0.35)	0.08
Moderate to vigorous physical activity (min/day)	50.63 (27.55)	36.59 (19.81)	0.09
Steps per day	6160.66 (2552.86)	5537.08 (2399.42)	0.49
**Dietary Intake at Baseline (mean (SD))**			
Total Energy (kcal/day)	1684.96 (558.52)	1950.87 (552.33)	0.18
Total Protein (g/day)	66.93 (24.40)	75.75 (21.46)	0.26
Total Fatty acids (g/day)	69.13 (28.43)	78.05 (25.29)	0.33
Saturated fatty acids (g/day)	22.61 (12.24)	25.39 (9.92)	0.46
Monounsaturated fatty acids (g/day)	22.90 (7.76)	27.85 (9.19)	0.11
Polyunsaturated fatty acids (g/day)	17.92 (8.61)	18.64 (7.81)	0.80
Cholesterol (mg/day)	211.18 (126.03)	296.05 (154.93)	0.10
Total HEI-2015 score[4]	55.16 (14.97)	52.00 (14.18)	0.53
**Maternal pregnancy complications**			
Gestational diabetes (%)	2 (16.7)	1 (3.7)	0.45
Gestational hypertension (%)	3 (25.0)	5 (18.5)	0.97
**Offspring conditions**			
Girl (%)	6 (50.0)	13 (48.1)	1
Low birth weight (%)[5]	0 (0.0)	2 (7.4)	0.49
Preterm birth (%)[5]	0 (0.0)	1 (3.7)	0.63
Small for gestational age (%)[5]	1 (8.3)	3 (11.1)	0.76
Large for gestational age (%)[5]	1 (8.3)	1 (8.3)	0.76

[1] Data are presented as frequency (percentage) for categorical variables and mean (standard deviations, SD) for continuous variables.

[2] Excessive GWG was defined using the 2009 IOM criteria as total GWG over 11.5 kg for women with prepregnancy BMI between 25-29.9 kg/m^2 or total GWG over 9 kg for women with prepregnancy BMI of 30 kg/m^2 or above.

[3] P-values were calculated using t-tests for continuous variables and Fisher's exact test for categorical variables.

[4] The HEI-2015 scores range from 0 to 100, with a higher HEI score reflecting better adherence to the 2015-2020 Dietary Guidelines for Americans.

[5] One offspring had missing data on low birth weight, preterm birth, small for gestational age, and large for gestational age.

Abbreviations: BMI, body mass index; HEI, healthy eating index; SD, standard deviation.

Table S3. Associations of 104 metabolites at baseline around 12 weeks of gestation with gestational weight gain in linear regression analyses.

			Unadjusted			Adjusted[1]		
**Metabolite**	**Superclass**	**Class**	β	**P**	**FDR**	β	**P**	**FDR**
Arachidonyl dopamine	Benzenoids	Phenols	-4.01	0.03	0.36	-3.86	0.05	0.37
3-Hydroxybutyrylcarnitine	Lipids and lipid-like molecules	Fatty Acyls	-4.87	<0.001	0.07	-4.94	<0.001	0.15
AC (18:2)	Lipids and lipid-like molecules	Fatty Acyls	-3.90	0.003	0.21	-4.61	0.001	0.16
O-Acetyl-L-carnitine	Lipids and lipid-like molecules	Fatty Acyls	-4.13	0.002	0.21	-4.46	0.001	0.16
AC (10:1)	Lipids and lipid-like molecules	Fatty Acyls	-4.04	0.002	0.21	-4.40	0.003	0.16
AC (18:1)	Lipids and lipid-like molecules	Fatty Acyls	-3.74	0.01	0.23	-4.58	0.002	0.16
Adipic acid	Lipids and lipid-like molecules	Fatty Acyls	3.59	0.01	0.24	4.23	0.003	0.16
Acetylcarnitine	Lipids and lipid-like molecules	Fatty Acyls	-3.85	0.004	0.21	-4.22	0.003	0.16
AC (14:1)	Lipids and lipid-like molecules	Fatty Acyls	-3.83	0.004	0.21	-4.08	0.005	0.21
AC (12:1)	Lipids and lipid-like molecules	Fatty Acyls	-3.51	0.01	0.29	-4.62	0.01	0.23
AC (14:2)	Lipids and lipid-like molecules	Fatty Acyls	-3.69	0.01	0.23	-3.95	0.01	0.23
Hexanoyl-L-carnitine	Lipids and lipid-like molecules	Fatty Acyls	-3.20	0.02	0.30	-3.61	0.01	0.25
AC (10:0)	Lipids and lipid-like molecules	Fatty Acyls	-3.38	0.01	0.29	-3.39	0.02	0.27
Octanoylcarnitine	Lipids and lipid-like molecules	Fatty Acyls	-3.12	0.02	0.31	-3.16	0.03	0.33
AC (16:0)	Lipids and lipid-like molecules	Fatty Acyls	-2.55	0.06	0.42	-3.01	0.04	0.36
AC (18:0)	Lipids and lipid-like molecules	Fatty Acyls	-2.82	0.04	0.36	-2.87	0.04	0.36
3-Hydroxyoleylcarnitine	Lipids and lipid-like molecules	Fatty Acyls	-2.35	0.09	0.46	-3.00	0.04	0.36
Decanoyl-L-carnitine	Lipids and lipid-like molecules	Fatty Acyls	-3.09	0.02	0.31	-3.05	0.04	0.36
D-Turanose	Lipids and lipid-like molecules	Fatty Acyls	2.68	0.05	0.37	2.86	0.05	0.36
Heptadecanoic acid	Lipids and lipid-like molecules	Fatty Acyls	-3.29	0.02	0.29	-3.01	0.05	0.37
(R)-Butyrylcarnitine	Lipids and lipid-like molecules	Fatty Acyls	-2.29	0.10	0.46	-2.98	0.05	0.37
TAG 43:0 or TAG 13:0-14:0-16:0	Lipids and lipid-like molecules	Glycerolipids	3.80	0.004	0.21	3.84	0.01	0.23
TAG (44:0)	Lipids and lipid-like molecules	Glycerolipids	3.79	0.004	0.22	3.69	0.01	0.25
TAG (42:1)	Lipids and lipid-like molecules	Glycerolipids	3.63	0.01	0.24	3.70	0.01	0.25
TAG (42:0)	Lipids and lipid-like molecules	Glycerolipids	3.69	0.01	0.23	3.57	0.01	0.25
TAG (42:2)	Lipids and lipid-like molecules	Glycerolipids	3.54	0.01	0.27	3.46	0.02	0.27
TAG (44:1)	Lipids and lipid-like molecules	Glycerolipids	3.50	0.01	0.27	3.41	0.02	0.27
TAG 46:2 or TAG 12:0-16:1-18:1	Lipids and lipid-like molecules	Glycerolipids	3.42	0.01	0.28	3.33	0.02	0.28
TAG (46:1)	Lipids and lipid-like molecules	Glycerolipids	3.44	0.01	0.28	3.29	0.02	0.28
TAG (44:2)	Lipids and lipid-like molecules	Glycerolipids	3.35	0.01	0.29	3.35	0.03	0.30
TAG (52:6)	Lipids and lipid-like molecules	Glycerolipids	2.92	0.03	0.36	2.82	0.05	0.36
TAG 45:1 or TAG 12:0-16:0-17:1	Lipids and lipid-like molecules	Glycerolipids	3.07	0.02	0.33	3.01	0.04	0.36
TAG 47:2 or TAG 14:0-15:0-18:2	Lipids and lipid-like molecules	Glycerolipids	3.01	0.03	0.35	2.88	0.05	0.36
TAG (52:5)	Lipids and lipid-like molecules	Glycerolipids	2.84	0.04	0.36	2.85	0.04	0.36
TAG (46:0)	Lipids and lipid-like molecules	Glycerolipids	3.33	0.01	0.29	3.04	0.04	0.36
PC (28:0)	Lipids and lipid-like molecules	Glycerophospholipids	3.68	0.01	0.23	4.12	0.003	0.16
PC (30:0)	Lipids and lipid-like molecules	Glycerophospholipids	3.83	0.004	0.21	4.17	0.002	0.16
PC (36:1) A	Lipids and lipid-like molecules	Glycerophospholipids	3.74	0.005	0.23	4.38	0.003	0.16
PE (p-38:2) or PE (o-38:3)	Lipids and lipid-like molecules	Glycerophospholipids	4.02	0.002	0.21	4.29	0.003	0.16
PC (34:0) A	Lipids and lipid-like molecules	Glycerophospholipids	3.29	0.01	0.29	4.15	0.005	0.21
PC (34:4)	Lipids and lipid-like molecules	Glycerophospholipids	3.41	0.01	0.28	3.74	0.01	0.24
PC (40:6) A	Lipids and lipid-like molecules	Glycerophospholipids	3.15	0.02	0.30	3.80	0.01	0.25
LPC (14:0) A	Lipids and lipid-like molecules	Glycerophospholipids	2.89	0.03	0.36	3.82	0.02	0.25
LPC (14:0) B	Lipids and lipid-like molecules	Glycerophospholipids	2.91	0.03	0.36	3.88	0.01	0.25
LPE (18:0)	Lipids and lipid-like molecules	Glycerophospholipids	2.87	0.03	0.36	4.05	0.02	0.25
PC (32:2) A	Lipids and lipid-like molecules	Glycerophospholipids	2.91	0.03	0.36	3.56	0.02	0.25
PC (34:4)	Lipids and lipid-like molecules	Glycerophospholipids	3.13	0.02	0.30	3.51	0.01	0.25
PC (31:1) A	Lipids and lipid-like molecules	Glycerophospholipids	2.77	0.04	0.36	3.79	0.01	0.25
PC (40:7) A	Lipids and lipid-like molecules	Glycerophospholipids	3.30	0.01	0.29	3.64	0.01	0.25
PC (38:5) A	Lipids and lipid-like molecules	Glycerophospholipids	3.30	0.01	0.29	3.67	0.01	0.25
PC (31:0)	Lipids and lipid-like molecules	Glycerophospholipids	2.62	0.05	0.39	3.80	0.01	0.25
PC (33:0)	Lipids and lipid-like molecules	Glycerophospholipids	2.39	0.08	0.46	3.59	0.01	0.25
PC (42:6)	Lipids and lipid-like molecules	Glycerophospholipids	3.29	0.01	0.29	3.85	0.01	0.25
PC (38:2) A	Lipids and lipid-like molecules	Glycerophospholipids	3.40	0.01	0.28	3.49	0.01	0.25
PC (36:6)	Lipids and lipid-like molecules	Glycerophospholipids	2.70	0.05	0.37	3.48	0.02	0.26
PC (36:4) B	Lipids and lipid-like molecules	Glycerophospholipids	3.09	0.02	0.31	3.49	0.02	0.27
PC (33:1) B	Lipids and lipid-like molecules	Glycerophospholipids	2.49	0.07	0.42	3.42	0.02	0.27
PC (34:0) B	Lipids and lipid-like molecules	Glycerophospholipids	2.71	0.05	0.37	3.39	0.02	0.27
PC (36:1) B	Lipids and lipid-like molecules	Glycerophospholipids	2.94	0.03	0.36	3.43	0.02	0.27
PC (32:2) B	Lipids and lipid-like molecules	Glycerophospholipids	2.69	0.05	0.37	3.37	0.02	0.29
PC (32:1) A	Lipids and lipid-like molecules	Glycerophospholipids	2.89	0.03	0.36	3.25	0.03	0.33
PC 30:0e or PC 14:0e/16:0	Lipids and lipid-like molecules	Glycerophospholipids	1.82	0.19	0.58	3.42	0.03	0.33
PC (38:5) B	Lipids and lipid-like molecules	Glycerophospholipids	2.63	0.05	0.38	3.12	0.03	0.34
LPC (20:2)	Lipids and lipid-like molecules	Glycerophospholipids	2.51	0.06	0.42	3.27	0.05	0.36
PC (42:10)	Lipids and lipid-like molecules	Glycerophospholipids	2.05	0.14	0.52	3.26	0.04	0.36
PC (40:8)	Lipids and lipid-like molecules	Glycerophospholipids	2.28	0.10	0.46	3.01	0.05	0.36
PC (37:5)	Lipids and lipid-like molecules	Glycerophospholipids	2.12	0.12	0.49	3.06	0.04	0.36
PE (p-38:6) or PE (o-38:7)	Lipids and lipid-like molecules	lysophosphatidylcolines	2.74	0.05	0.37	3.05	0.03	0.36
PE (p-40:7) or PE (o-40:8)	Lipids and lipid-like molecules	lysophosphatidylcolines	2.61	0.06	0.42	2.97	0.04	0.36
PC (33:1)	Lipids and lipid-like molecules	Glycerophospholipids	2.39	0.08	0.46	2.93	0.04	0.36
PC 32:1e or PC 14:0e/18:1	Lipids and lipid-like molecules	Glycerophospholipids	1.99	0.16	0.55	3.31	0.04	0.36
PC (37:3)	Lipids and lipid-like molecules	Glycerophospholipids	2.02	0.14	0.53	3.20	0.04	0.36

PC (35:1)	Lipids and lipid-like molecules	Glycerophospholipids	2.46	0.07	0.43	2.97	0.04	0.36
PC (39:4)	Lipids and lipid-like molecules	Glycerophospholipids	2.12	0.13	0.50	3.22	0.04	0.36
PC (38:2) B	Lipids and lipid-like molecules	Glycerophospholipids	2.83	0.04	0.36	2.90	0.04	0.36
PC (32:1) B	Lipids and lipid-like molecules	Glycerophospholipids	2.69	0.05	0.37	2.91	0.05	0.37
SM (d30:1) A	Lipids and lipid-like molecules	Sphingolipids	3.94	0.003	0.21	4.98	<0.001	0.15
SM (d32:0) A	Lipids and lipid-like molecules	Sphingolipids	3.93	0.003	0.21	4.42	0.001	0.16
SM(d30:1) B	Lipids and lipid-like molecules	Sphingolipids	3.17	0.02	0.30	4.20	0.004	0.19
SM(d32:0) B	Lipids and lipid-like molecules	Sphingolipids	3.22	0.02	0.29	3.90	0.01	0.23
SM (d32:1) A	Lipids and lipid-like molecules	Sphingolipids	2.85	0.04	0.36	3.70	0.01	0.25
SM (d39:1)	Lipids and lipid-like molecules	Sphingolipids	2.43	0.08	0.44	3.70	0.02	0.25
Ceramide (d32:1)	Lipids and lipid-like molecules	Sphingolipids	2.94	0.03	0.36	3.45	0.02	0.26
SM (d41:1)	Lipids and lipid-like molecules	Sphingolipids	2.22	0.10	0.47	3.19	0.03	0.33
SM (d32:1) B	Lipids and lipid-like molecules	Sphingolipids	2.32	0.09	0.46	3.12	0.03	0.36
Ceramide (d39:1)	Lipids and lipid-like molecules	Sphingolipids	2.36	0.09	0.46	3.21	0.04	0.36
GlcCer (d41:1)	Lipids and lipid-like molecules	Sphingolipids	2.14	0.12	0.49	2.95	0.04	0.36
CE (18:3)	Lipids and lipid-like molecules	Steroids and steroid derivatives	2.78	0.04	0.36	3.59	0.02	0.27
CE (16:1)	Lipids and lipid-like molecules	Steroids and steroid derivatives	2.64	0.05	0.39	3.15	0.04	0.36
Citric acid	Organic acids and derivatives	Carboxylic acids and derivatives	-3.38	0.01	0.29	-4.22	0.003	0.16
Betaine	Organic acids and derivatives	Carboxylic acids and derivatives	-3.14	0.02	0.30	-3.18	0.02	0.29
Creatine	Organic acids and derivatives	Carboxylic acids and derivatives	3.28	0.01	0.29	3.34	0.02	0.29
Aconitic acid	Organic acids and derivatives	Carboxylic acids and derivatives	-2.20	0.11	0.47	-3.18	0.03	0.36
3-hydroxybutyric acid	Organic acids and derivatives	Hydroxy acids and derivatives	-3.59	0.01	0.27	-4.28	0.01	0.23
L-Carnitine	Organic nitrogen compounds	Organonitrogen compounds	-3.31	0.01	0.29	-3.71	0.01	0.23
Carnitine	Organic nitrogen compounds	Organonitrogen compounds	-3.24	0.02	0.29	-3.63	0.01	0.25
SM (d32:2) A	Lipids and lipid-like molecules	Sphingolipids	2.93	0.04	0.36	3.65	0.02	0.25
SM (d32:2) B	Lipids and lipid-like molecules	Sphingolipids	2.62	0.06	0.42	3.45	0.02	0.29
SM (d41:2) B	Lipids and lipid-like molecules	Sphingolipids	1.84	0.18	0.57	3.03	0.05	0.37
1-methylgalactose	Organic oxygen compounds	Organooxygen compounds	3.95	0.00	0.21	3.74	0.01	0.25
Threitol	Organic oxygen compounds	Organooxygen compounds	3.15	0.02	0.30	3.63	0.05	0.36
4-Imidazoleacrylic acid	Organoheterocyclic compounds	Azoles	-3.10	0.02	0.31	-3.38	0.02	0.27
Serotonin	Organoheterocyclic compounds	Indoles and derivatives	4.29	0.002	0.21	4.44	0.01	0.23
Trans-3'-Hydroxycotinine	Organoheterocyclic compounds	Pyridines and derivatives	4.82	<0.001	0.07	5.14	0.001	0.16

[1] Multivariable linear regression models adjusted for age, race, parity, and prepregnancy BMI. Multiple comparisons were adjusted using the Benjamini-Hochberg procedure, with FDR <0.05 as the statistically significant level.

Abbreviation: AC, acylcarnitines; BMI, body mass index; CE, cholesteryl esters; GlcCer, glucosylceramide; GWG, gestational weight gain; LPC, lysophosphatidylcolines; PC, phosphatidylcholine; PE, phosphatidylethanolamine; SM, sphingomyelin; TAG, triacylglycerol.

Table S4. Associations of 186 metabolites at 32 weeks of gestation with gestational weight gain in linear regression analyses.

Metabolite	Superclass	Class	Unadjusted			Adjusted[1]		
			β	P	FDR	β	P	FDR
PC (34:4) A	Lipids and lipid-like molecules	Glycerophospholipids	3.97	0.002	0.11	6.00	<0.001	0.001
PC (34:4) B	Lipids and lipid-like molecules	Glycerophospholipids	4.03	0.002	0.10	5.94	<0.001	0.001
TAG (52:6)	Lipids and lipid-like molecules	Glycerolipids	4.26	<0.001	0.09	5.46	<0.001	0.001
PC (36:4) B	Lipids and lipid-like molecules	Glycerophospholipids	3.44	0.01	0.13	5.95	<0.001	0.001
PC (34:3) C	Lipids and lipid-like molecules	Glycerophospholipids	3.61	0.004	0.13	6.06	<0.001	0.001
PC (36:6)	Lipids and lipid-like molecules	Glycerophospholipids	3.06	0.02	0.20	5.83	<0.001	0.002
PC (31:1) A	Lipids and lipid-like molecules	Glycerophospholipids	3.01	0.02	0.20	5.85	<0.001	0.002
PC (30:0)	Lipids and lipid-like molecules	Glycerophospholipids	3.85	0.002	0.10	5.08	<0.001	0.003
TAG (54:7) B	Lipids and lipid-like molecules	Glycerolipids	3.79	0.002	0.10	5.29	<0.001	0.004
TAG (52:5)	Lipids and lipid-like molecules	Glycerolipids	4.15	0.001	0.09	4.78	<0.001	0.004
PC (34:3)	Lipids and lipid-like molecules	Glycerophospholipids	3.53	0.01	0.13	5.22	<0.001	0.004
TAG (50:5)	Lipids and lipid-like molecules	Glycerolipids	4.11	0.001	0.09	4.91	<0.001	0.005
TAG 47:2 or TAG 14:0-15:0-18:2	Lipids and lipid-like molecules	Glycerolipids	3.45	0.01	0.13	4.88	<0.001	0.01
PC (34:3) B	Lipids and lipid-like molecules	Glycerophospholipids	3.72	0.003	0.13	5.00	<0.001	0.01
TAG (48:2)	Lipids and lipid-like molecules	Glycerolipids	3.69	0.003	0.11	4.89	<0.001	0.01
TAG (58:9)	Lipids and lipid-like molecules	Glycerolipids	4.50	0.001	0.09	5.53	<0.001	0.01
TAG (54:7) A	Lipids and lipid-like molecules	Glycerolipids	4.12	0.001	0.09	4.91	<0.001	0.01
TAG 52:6 or TAG 14:0-18:2-20:4	Lipids and lipid-like molecules	Glycerolipids	3.65	0.004	0.13	4.92	<0.001	0.01
LPC (14:0)	Lipids and lipid-like molecules	Glycerophospholipids	3.40	0.01	0.16	5.01	<0.001	0.01
PC (32:1) A	Lipids and lipid-like molecules	Glycerophospholipids	3.17	0.01	0.17	5.08	<0.001	0.01
PC (32:1) B	Lipids and lipid-like molecules	Glycerophospholipids	3.24	0.01	0.16	5.07	<0.001	0.01
PC (32:2)	Lipids and lipid-like molecules	Glycerophospholipids	2.95	0.03	0.22	4.94	<0.001	0.01
TAG 47:1 or TAG 15:0-16:0-16:1	Lipids and lipid-like molecules	Glycerolipids	3.56	0.01	0.13	4.71	<0.001	0.01
PC (40:7) A	Lipids and lipid-like molecules	Glycerophospholipids	2.85	0.03	0.23	5.26	<0.001	0.01
TAG (48:5)	Lipids and lipid-like molecules	Glycerolipids	3.90	0.002	0.10	4.54	<0.001	0.01
TAG 58:7 or TAG 18:0-18:2-22:5	Lipids and lipid-like molecules	Glycerolipids	3.93	0.001	0.09	4.91	<0.001	0.01
TAG (46:1)	Lipids and lipid-like molecules	Glycerolipids	3.56	0.004	0.13	4.72	<0.001	0.01
TAG (53:5)	Lipids and lipid-like molecules	Glycerolipids	3.66	0.004	0.13	4.87	<0.001	0.01
TAG (52:4)	Lipids and lipid-like molecules	Glycerolipids	4.20	<0.001	0.09	4.71	<0.001	0.01
TAG (54:8)	Lipids and lipid-like molecules	Glycerolipids	3.17	0.01	0.18	4.75	<0.001	0.01
TAG (48:1)	Lipids and lipid-like molecules	Glycerolipids	3.80	0.002	0.10	4.57	<0.001	0.01
PC (28:0)	Lipids and lipid-like molecules	Glycerophospholipids	3.19	0.01	0.18	4.60	<0.001	0.01
TAG (48:4) B	Lipids and lipid-like molecules	Glycerolipids	3.96	0.001	0.09	4.52	<0.001	0.01
TAG 49:3 or TAG 15:0-16:1-18:2	Lipids and lipid-like molecules	Glycerolipids	2.81	0.03	0.23	4.78	<0.001	0.01
LPC (14:0)	Lipids and lipid-like molecules	Glycerophospholipids	2.76	0.04	0.25	4.88	<0.001	0.01
PC (32:2)	Lipids and lipid-like molecules	Glycerophospholipids	2.91	0.03	0.23	4.57	0.001	0.01
SM (d30:1) A	Lipids and lipid-like molecules	Sphingolipids	3.12	0.01	0.18	4.50	0.001	0.01
PC (40:8)	Lipids and lipid-like molecules	Glycerophospholipids	2.76	0.04	0.26	5.71	0.001	0.01
PC (38:3)	Lipids and lipid-like molecules	Glycerophospholipids	2.97	0.02	0.20	5.08	0.001	0.01
TAG (46:0)	Lipids and lipid-like molecules	Glycerolipids	3.96	0.001	0.09	4.08	0.001	0.01
TAG (56:9)	Lipids and lipid-like molecules	Glycerolipids	3.23	0.01	0.17	4.45	0.001	0.01
PC (38:6) A	Lipids and lipid-like molecules	Glycerophospholipids	3.26	0.02	0.20	5.33	0.001	0.02
TAG (44:0)	Lipids and lipid-like molecules	Glycerolipids	3.62	0.004	0.13	4.19	0.001	0.02
PC (36:5) C	Lipids and lipid-like molecules	Glycerophospholipids	2.45	0.06	0.30	4.69	0.001	0.02
TAG (49:2)	Lipids and lipid-like molecules	Glycerolipids	2.93	0.02	0.20	4.49	0.001	0.02
PC (38:4) B	Lipids and lipid-like molecules	Glycerophospholipids	3.22	0.01	0.17	4.24	0.001	0.02
PC (36:3) A	Lipids and lipid-like molecules	Glycerophospholipids	2.25	0.08	0.34	5.37	0.001	0.02
Isoleucine	Organic acids and derivatives	Carboxylic acids and derivatives	3.97	0.001	0.09	4.10	0.001	0.02
PC (33:1)	Lipids and lipid-like molecules	Glycerophospholipids	2.60	0.05	0.28	4.89	0.001	0.02
SM (d30:1) B	Lipids and lipid-like molecules	Sphingolipids	2.72	0.03	0.24	4.29	0.001	0.02
TAG (50:3) A	Lipids and lipid-like molecules	Glycerolipids	2.66	0.04	0.26	4.63	0.001	0.02
TAG (44:1)	Lipids and lipid-like molecules	Glycerolipids	3.26	0.01	0.18	4.21	0.001	0.02
PC (36:5) D	Lipids and lipid-like molecules	Glycerophospholipids	2.71	0.03	0.24	4.81	0.001	0.02
SM (d32:2)	Lipids and lipid-like molecules	Sphingolipids	2.73	0.03	0.24	4.31	0.002	0.02
PC (36:3) B	Lipids and lipid-like molecules	Glycerophospholipids	2.46	0.06	0.29	5.15	0.002	0.02
TAG 46:2 or TAG 12:0-16:1-18:1	Lipids and lipid-like molecules	Glycerolipids	3.10	0.02	0.19	4.05	0.002	0.03
PC (38:7)	Lipids and lipid-like molecules	Glycerophospholipids	1.58	0.23	0.56	5.10	0.002	0.03

PC (36:5) B	Lipids and lipid-like molecules	Glycerophospholipids	2.79	0.03	0.23	4.48	0.002	0.03
TAG (54:5) B	Lipids and lipid-like molecules	Glycerolipids	3.55	0.005	0.13	4.36	0.002	0.03
TAG 48:3 or TAG 14:0-16:1-18:2	Lipids and lipid-like molecules	Glycerolipids	2.59	0.04	0.27	4.18	0.002	0.03
PC (38:6) C	Lipids and lipid-like molecules	Glycerophospholipids	2.50	0.05	0.29	4.97	0.002	0.03
SM (d32:2)	Lipids and lipid-like molecules	Sphingolipids	2.31	0.08	0.33	4.31	0.002	0.03
PC (40:6) A	Lipids and lipid-like molecules	Glycerophospholipids	1.81	0.19	0.52	4.61	0.003	0.03
Phe-Trp	Organic acids and derivatives	Carboxylic acids and derivatives	4.51	0.001	0.09	4.77	0.003	0.03
PC (42:6)	Lipids and lipid-like molecules	Glycerophospholipids	2.52	0.06	0.30	4.70	0.003	0.03
TAG (48:3)	Lipids and lipid-like molecules	Glycerolipids	3.04	0.02	0.19	4.41	0.003	0.03
TAG 45:1 or TAG 12:0-16:0-17:1	Lipids and lipid-like molecules	Glycerolipids	2.90	0.02	0.22	3.86	0.003	0.03
Arachidonic acid	Lipids and lipid-like molecules	Fatty Acyls	3.17	0.01	0.17	3.75	0.003	0.03
PC (31:0)	Lipids and lipid-like molecules	Glycerophospholipids	2.07	0.12	0.44	4.58	0.003	0.03
PC (33:1) B	Lipids and lipid-like molecules	Glycerophospholipids	2.34	0.08	0.34	4.43	0.003	0.03
TAG (54:6) B	Lipids and lipid-like molecules	Glycerolipids	3.51	0.01	0.13	4.17	0.003	0.03
TAG (40:1)	Lipids and lipid-like molecules	Glycerolipids	3.44	0.01	0.13	3.75	0.003	0.03
TAG (44:2)	Lipids and lipid-like molecules	Glycerolipids	3.08	0.02	0.20	3.84	0.003	0.03
LPC (22:5)	Lipids and lipid-like molecules	Glycerophospholipids	3.67	0.003	0.11	3.66	0.003	0.03
TAG (46:3) A	Lipids and lipid-like molecules	Glycerolipids	2.80	0.03	0.23	3.80	0.003	0.04
LPC (16:1)	Lipids and lipid-like molecules	Glycerophospholipids	2.14	0.10	0.38	4.61	0.004	0.04
LPC (20:3)	Lipids and lipid-like molecules	Glycerophospholipids	2.78	0.03	0.23	4.41	0.004	0.04
TAG 45:0 or TAG 14:0-15:0-16:0	Lipids and lipid-like molecules	Glycerolipids	3.18	0.02	0.18	3.69	0.004	0.04
SM (d32:0)	Lipids and lipid-like molecules	Sphingolipids	2.18	0.10	0.38	4.03	0.004	0.04
PC (33:0)	Lipids and lipid-like molecules	Glycerophospholipids	2.35	0.08	0.34	4.18	0.004	0.04
TAG 50:5 or TAG 14:1-18:2-18:2	Lipids and lipid-like molecules	Glycerolipids	2.43	0.06	0.30	3.85	0.004	0.04
PC (35:4)	Lipids and lipid-like molecules	Glycerophospholipids	1.83	0.21	0.55	4.52	0.004	0.04
LPC (16:1)	Lipids and lipid-like molecules	Glycerophospholipids	2.45	0.06	0.30	4.36	0.004	0.04
LPC (20:3)	Lipids and lipid-like molecules	Glycerophospholipids	2.97	0.02	0.20	4.29	0.005	0.04
PC (37:5)	Lipids and lipid-like molecules	Glycerophospholipids	2.01	0.12	0.44	4.31	0.005	0.04
TAG (49:3)	Lipids and lipid-like molecules	Glycerolipids	2.31	0.07	0.33	4.06	0.005	0.04
TAG (50:2)	Lipids and lipid-like molecules	Glycerolipids	3.15	0.01	0.17	3.91	0.01	0.04
PC (34:3) A	Lipids and lipid-like molecules	Glycerophospholipids	1.40	0.29	0.61	4.58	0.01	0.05
TAG (42:3)	Lipids and lipid-like molecules	Glycerolipids	3.31	0.01	0.16	3.71	0.01	0.05
PC (36:1)	Lipids and lipid-like molecules	Glycerophospholipids	3.25	0.01	0.18	4.05	0.01	0.06
TAG (46:4) B	Lipids and lipid-like molecules	Glycerolipids	3.34	0.01	0.14	3.74	0.01	0.06
PE 40:6 or PE 20:3-20:3	Lipids and lipid-like molecules	Glycerophospholipids	3.48	0.005	0.13	4.13	0.01	0.06
Glyceric acid	Organic oxygen compounds	Organooxygen compounds	3.16	0.01	0.18	3.52	0.01	0.06
TAG 56:7 or TAG 16:0-18:1-22:6	Lipids and lipid-like molecules	Glycerolipids	2.92	0.02	0.20	3.83	0.01	0.07
PC (40:4)	Lipids and lipid-like molecules	Glycerophospholipids	3.31	0.01	0.16	3.32	0.01	0.07
TAG (42:0)	Lipids and lipid-like molecules	Glycerolipids	2.89	0.03	0.23	3.40	0.01	0.07
Glycerol-alpha-phosphate	Lipids and lipid-like molecules	Glycerophospholipids	3.18	0.01	0.17	3.23	0.01	0.08
LPC (15:0)	Lipids and lipid-like molecules	Glycerophospholipids	0.91	0.55	0.79	5.10	0.01	0.08
TAG (49:1)	Lipids and lipid-like molecules	Glycerolipids	2.87	0.02	0.22	3.46	0.01	0.08
TAG 50:4 or TAG 16:1-16:1-18:2	Lipids and lipid-like molecules	Glycerolipids	2.13	0.10	0.39	3.56	0.01	0.08
TAG (55:1)	Lipids and lipid-like molecules	Glycerolipids	3.48	0.01	0.13	3.49	0.01	0.08
LPC (22:5)	Lipids and lipid-like molecules	Glycerophospholipids	3.13	0.01	0.18	3.33	0.01	0.08
SM (d32:0)	Lipids and lipid-like molecules	Sphingolipids	1.64	0.22	0.55	3.68	0.01	0.08
PC (34:1)	Lipids and lipid-like molecules	Glycerophospholipids	3.07	0.02	0.19	3.61	0.01	0.08
PC (37:3)	Lipids and lipid-like molecules	Glycerophospholipids	1.13	0.40	0.70	4.33	0.01	0.08
PC (40:5) A	Lipids and lipid-like molecules	Glycerophospholipids	3.12	0.01	0.18	3.31	0.01	0.08
TAG (56:8) A	Lipids and lipid-like molecules	Glycerolipids	2.78	0.03	0.23	3.51	0.01	0.08
Ceramide (d39:1)	Lipids and lipid-like molecules	Sphingolipids	1.74	0.21	0.55	4.06	0.01	0.08
SM (d32:1)	Lipids and lipid-like molecules	Sphingolipids	2.03	0.12	0.43	3.61	0.01	0.09
3-Hydroxybutyrylcarnitine	Lipids and lipid-like molecules	Fatty Acyls	-2.56	0.06	0.30	-3.32	0.01	0.09
PE (p-40:7) or PE (o-40:8)	Lipids and lipid-like molecules	Glycerophospholipids	1.93	0.14	0.46	4.02	0.01	0.09
CE (18:2)	Lipids and lipid-like molecules	Steroids and steroid derivatives	-3.43	0.01	0.13	-3.43	0.01	0.09
LPE (22:6)	Lipids and lipid-like molecules	Glycerophospholipids	3.28	0.01	0.16	3.27	0.01	0.10
Hydroxycarbamate	Organic acids and derivatives	Organic carbonic acids and derivatives	2.88	0.02	0.21	3.18	0.01	0.10
TAG (50:4)	Lipids and lipid-like molecules	Glycerolipids	2.01	0.12	0.44	3.35	0.02	0.10
TAG (56:5) B	Lipids and lipid-like molecules	Glycerolipids	2.97	0.02	0.20	3.39	0.02	0.10
PC (36:1)	Lipids and lipid-like molecules	Glycerophospholipids	2.82	0.03	0.23	3.57	0.02	0.10
PE (p-34:2) or PE (o-34:3)	Lipids and lipid-like molecules	Glycerophospholipids	2.26	0.08	0.34	3.20	0.02	0.10

PC (35:1)	Lipids and lipid-like molecules	Glycerophospholipids	2.54	0.06	0.30	3.67	0.02	0.11
TAG (57:2)	Lipids and lipid-like molecules	Glycerolipids	3.04	0.02	0.19	3.39	0.02	0.11
LPC (20:5)	Lipids and lipid-like molecules	Glycerophospholipids	2.05	0.12	0.43	3.88	0.02	0.11
PC (42:10)	Lipids and lipid-like molecules	Glycerophospholipids	1.42	0.29	0.62	4.32	0.02	0.11
Sorbitol	Organic oxygen compounds	Organooxygen compounds	2.76	0.03	0.23	3.12	0.02	0.11
SM (d32:1)	Lipids and lipid-like molecules	Sphingolipids	1.80	0.17	0.50	3.43	0.02	0.11
SM (d39:1)	Lipids and lipid-like molecules	Sphingolipids	1.12	0.43	0.73	4.10	0.02	0.11
Leucine	Organic acids and derivatives	Carboxylic acids and derivatives	3.83	0.00	0.13	3.25	0.02	0.11
TAG (48:4) A	Lipids and lipid-like molecules	Glycerolipids	1.71	0.19	0.53	3.36	0.02	0.11
PC (40:4)	Lipids and lipid-like molecules	Glycerophospholipids	3.03	0.02	0.20	2.94	0.02	0.11
Betaine	Organic acids and derivatives	Carboxylic acids and derivatives	-2.91	0.02	0.20	-2.91	0.02	0.12
SM (d39:1)	Lipids and lipid-like molecules	Sphingolipids	1.01	0.48	0.76	3.95	0.02	0.12
PE (36:1)	Lipids and lipid-like molecules	Glycerophospholipids	2.53	0.05	0.29	3.43	0.02	0.12
TAG (56:7)	Lipids and lipid-like molecules	Glycerolipids	2.48	0.06	0.30	3.42	0.02	0.12
TAG (43:0)	Lipids and lipid-like molecules	Glycerolipids	2.65	0.04	0.27	2.95	0.02	0.12
TAG (49:0)	Lipids and lipid-like molecules	Glycerolipids	2.80	0.03	0.23	2.93	0.02	0.13
PC 37:5e or PC 18:5e/19:0	Lipids and lipid-like molecules	Glycerophospholipids	1.89	0.16	0.49	3.25	0.02	0.13
TAG (46:3) B	Lipids and lipid-like molecules	Glycerolipids	3.63	0.003	0.12	3.07	0.02	0.13
PC (34:1)	Lipids and lipid-like molecules	Glycerophospholipids	2.38	0.07	0.31	3.38	0.02	0.13
TAG (42:1)	Lipids and lipid-like molecules	Glycerolipids	2.57	0.06	0.29	3.03	0.02	0.14
GlcCer (d34:1)	Lipids and lipid-like molecules	Fatty Acyls	-3.04	0.02	0.20	-3.29	0.02	0.14
DG (38:5)	Lipids and lipid-like molecules	Glycerolipids	3.05	0.02	0.20	3.05	0.03	0.14
PC (40:6) B	Lipids and lipid-like molecules	Glycerophospholipids	1.98	0.13	0.44	3.29	0.03	0.14
PE (p-38:6) or PE (o-38:7)	Lipids and lipid-like molecules	Glycerophospholipids	1.80	0.17	0.49	3.32	0.03	0.14
PC (p-38:5) or PC(o-38:6) A	Lipids and lipid-like molecules	Glycerophospholipids	2.56	0.05	0.28	3.29	0.03	0.14
LPE (18:0)	Lipids and lipid-like molecules	Glycerophospholipids	3.14	0.01	0.18	3.30	0.03	0.14
PE (34:2)	Lipids and lipid-like molecules	Glycerophospholipids	2.37	0.07	0.31	3.27	0.03	0.15
CE (16:1)	Lipids and lipid-like molecules	Steroids and steroid derivatives	1.15	0.38	0.70	3.57	0.03	0.15
PC (40:5) A	Lipids and lipid-like molecules	Glycerophospholipids	2.47	0.06	0.29	2.84	0.03	0.15
PC (40:5) B	Lipids and lipid-like molecules	Glycerophospholipids	2.19	0.11	0.39	3.07	0.03	0.15
PC (32:0)	Lipids and lipid-like molecules	Glycerophospholipids	2.58	0.04	0.27	2.98	0.03	0.15
Erythritol	Organic oxygen compounds	Organooxygen compounds	2.55	0.05	0.28	2.81	0.03	0.15
Cer (d41:1)	Lipids and lipid-like molecules	Sphingolipids	1.87	0.16	0.49	3.30	0.03	0.15
PC (38:5) B	Lipids and lipid-like molecules	Glycerophospholipids	1.80	0.19	0.52	3.30	0.03	0.15
PC (38:2)	Lipids and lipid-like molecules	Glycerophospholipids	1.70	0.20	0.53	3.37	0.03	0.15
SM (d42:3)	Lipids and lipid-like molecules	Sphingolipids	-3.26	0.02	0.20	-3.10	0.03	0.16
TAG 47:0 or TAG 15:0-16:0-16:0	Lipids and lipid-like molecules	Glycerolipids	2.57	0.05	0.29	2.94	0.03	0.16
PC (p-40:5) or PC (o-40:6)	Lipids and lipid-like molecules	Glycerophospholipids	2.51	0.05	0.29	3.15	0.03	0.16
PC (38:5) A	Lipids and lipid-like molecules	Glycerophospholipids	2.15	0.10	0.39	2.99	0.03	0.16
PC (38:4) A	Lipids and lipid-like molecules	Glycerophospholipids	1.37	0.30	0.63	3.64	0.03	0.17
PI (34:1)	Lipids and lipid-like molecules	Glycerophospholipids	1.81	0.17	0.49	3.45	0.03	0.17
Ceramide (d41:1)	Lipids and lipid-like molecules	Sphingolipids	1.49	0.26	0.60	3.25	0.04	0.17
PC (38:5) A	Lipids and lipid-like molecules	Glycerophospholipids	2.42	0.07	0.32	3.06	0.04	0.17
PE (36:3)	Lipids and lipid-like molecules	Glycerophospholipids	1.99	0.13	0.44	3.15	0.04	0.17
LPC (17:1)	Lipids and lipid-like molecules	Glycerophospholipids	2.32	0.08	0.34	3.32	0.04	0.18
LPE (20:4)	Lipids and lipid-like molecules	Glycerophospholipids	3.03	0.02	0.20	2.92	0.04	0.18
PE (38:6)	Lipids and lipid-like molecules	Glycerophospholipids	2.59	0.04	0.27	3.08	0.04	0.18
PE (36:1)	Lipids and lipid-like molecules	Glycerophospholipids	3.46	0.01	0.13	3.15	0.04	0.18
PC (37:6)	Lipids and lipid-like molecules	Glycerophospholipids	0.28	0.85	0.95	3.94	0.04	0.18
SM (d41:1)	Lipids and lipid-like molecules	Sphingolipids	0.64	0.64	0.85	3.60	0.04	0.18
TAG (57:1)	Lipids and lipid-like molecules	Glycerolipids	3.07	0.02	0.20	3.12	0.04	0.18
PC 39:2e or PC 16:2e/23:0	Lipids and lipid-like molecules	Glycerophospholipids	-2.89	0.03	0.23	-2.83	0.04	0.18
3-Hydroxyphenylacetic acid	Benzenoids	Phenols	-2.30	0.07	0.33	-2.75	0.04	0.18
Stearic acid	Lipids and lipid-like molecules	Fatty Acyls	-2.61	0.04	0.27	-2.60	0.04	0.19
TAG 48:4 or TAG 12:0-18:2-18:2	Lipids and lipid-like molecules	Glycerolipids	1.79	0.18	0.51	2.81	0.04	0.19
PC (38:5) B	Lipids and lipid-like molecules	Glycerophospholipids	1.34	0.34	0.67	3.17	0.04	0.19
TAG (58:1)	Lipids and lipid-like molecules	Glycerolipids	2.87	0.03	0.23	3.02	0.04	0.19
SM (d39:2)	Lipids and lipid-like molecules	Sphingolipids	1.25	0.34	0.68	3.15	0.04	0.19
PE (38:6)	Lipids and lipid-like molecules	Glycerophospholipids	2.72	0.03	0.24	3.31	0.04	0.19
SM (d38:2)	Lipids and lipid-like molecules	Sphingolipids	-3.58	0.01	0.14	-2.98	0.05	0.20
LPE (16:0)	Lipids and lipid-like molecules	Glycerophospholipids	2.90	0.02	0.20	2.88	0.05	0.20
TAG (58:6)	Lipids and lipid-like molecules	Glycerolipids	2.53	0.05	0.29	2.78	0.05	0.20
TAG (42:2)	Lipids and lipid-like molecules	Glycerolipids	2.42	0.07	0.32	2.71	0.05	0.20
PC (o-32:0)	Lipids and lipid-like molecules	Glycerophospholipids	1.95	0.14	0.46	2.96	0.05	0.20
TAG (62:4)	Lipids and lipid-like molecules	Glycerolipids	2.35	0.08	0.34	3.01	0.05	0.20

Mannitol	Organic oxygen compounds	Organooxygen compounds	2.24	0.08	0.34	2.89	0.05	0.20
PC (39:6)	Lipids and lipid-like molecules	Glycerophospholipids	0.23	0.87	0.96	3.54	0.05	0.20
TAG (56:1)	Lipids and lipid-like molecules	Glycerolipids	2.79	0.04	0.26	2.98	0.05	0.20

[1] Multivariable linear regression analyses adjusted for age, race, parity, and prepregnancy BMI. Multiple comparisons were adjusted using the Benjamini-Hochberg procedure, with FDR <0.05 as the statistically significant level.

Abbreviation: AC, acylcarnitines; BMI, body mass index; CE, cholesteryl esters; GlcCer, glucosylceramide; GWG, gestational weight gain; LPC, lysophosphatidylcolines; PC, phosphatidylcholine; PE, phosphatidylethanolamine; SM, sphingomyelin; TAG, triacylglycerol.

Table S5. Associations of 47 changes of metabolites from baseline to 32 weeks of gestation with gestational weight gain in linear regression analyses.

Metabolite	Superclass	Class	Unadjusted			Adjusted[1]		
			β	P	FDR	β	P	FDR
TAG 50:4 or TAG 16:1-16:1-18:2	Lipids and lipid-like molecules	Glycerolipids	0.60	0.03	0.83	0.78	0.004	0.26
TAG (50:3) A	Lipids and lipid-like molecules	Glycerolipids	0.13	0.02	0.82	0.17	0.002	0.26
TAG (62:3)	Lipids and lipid-like molecules	Glycerolipids	-0.69	0.05	0.85	-0.97	0.004	0.26
TAG 49:3 or TAG 15:0-16:1-18:2	Lipids and lipid-like molecules	Glycerolipids	-0.64	0.09	1.00	-0.92	0.01	0.42
TAG (58:5)	Lipids and lipid-like molecules	Glycerolipids	-1.43	0.09	1.00	-2.17	0.01	0.42
LPC (p-18:0) or LPC (o-18:1)	Lipids and lipid-like molecules	Glycerophospholipids	-0.46	0.03	0.85	-0.62	0.003	0.26
PC (36:4) A	Lipids and lipid-like molecules	Glycerophospholipids	0.54	0.02	0.82	0.70	0.002	0.26
PC (p-42:4) or PC (o-42:5)	Lipids and lipid-like molecules	Glycerophospholipids	0.34	0.03	0.83	0.45	0.004	0.26
PC (p-34:1) or PC (o-34:2)	Lipids and lipid-like molecules	Glycerophospholipids	0.31	0.06	0.94	0.46	0.01	0.30
PC (34:1)	Lipids and lipid-like molecules	Glycerophospholipids	0.45	<0.001	0.07	0.46	0.01	0.42
PC (35:2) A	Lipids and lipid-like molecules	Glycerophospholipids	0.82	0.03	0.85	1.02	0.01	0.42
PC (p-40:4) or PC (o-40:5)	Lipids and lipid-like molecules	Glycerophospholipids	0.60	0.07	0.96	0.84	0.01	0.42
LPC (16:1)	Lipids and lipid-like molecules	Glycerophospholipids	-1.53	0.15	1.00	-2.78	0.02	0.52
PC (32:2)	Lipids and lipid-like molecules	Glycerophospholipids	-0.43	0.14	1.00	-0.70	0.02	0.52
PC (p-34:1) or PC (o-34:2) A	Lipids and lipid-like molecules	Glycerophospholipids	0.34	0.02	0.77	0.33	0.02	0.53
PC (38:7)	Lipids and lipid-like molecules	Glycerophospholipids	0.16	0.09	1.00	0.22	0.02	0.55
PC (p-42:5) or PC (o-42:6) B	Lipids and lipid-like molecules	Glycerophospholipids	0.04	0.06	0.94	0.05	0.03	0.61
PC (36:2)	Lipids and lipid-like molecules	Glycerophospholipids	-0.19	0.09	1.00	-0.23	0.03	0.63
LPC (22:6)	Lipids and lipid-like molecules	Glycerophospholipids	-0.66	0.04	0.85	-0.71	0.04	0.72
PC (p-36:1) or PC (o-36:2) A	Lipids and lipid-like molecules	Glycerophospholipids	0.46	0.17	1.00	0.68	0.04	0.72
PC (40:8)	Lipids and lipid-like molecules	Glycerophospholipids	0.01	0.00	0.12	0.01	0.04	0.73
PE (38:6)	Lipids and lipid-like molecules	Glycerophospholipids	-0.38	0.18	1.00	-0.61	0.04	0.74
LPC (18:3)	Lipids and lipid-like molecules	Glycerophospholipids	-0.99	0.09	1.00	-1.15	0.05	0.78
PC (p-36:2) or PC (o-36:3)	Lipids and lipid-like molecules	Glycerophospholipids	0.38	0.11	1.00	0.47	0.04	0.78
PC (35:3)	Lipids and lipid-like molecules	Glycerophospholipids	0.25	0.08	1.00	0.30	0.05	0.81
Ceramide (d33:1)	Lipids and lipid-like molecules	Sphingolipids	-0.48	0.002	0.17	-0.48	0.002	0.26
SM (d43:1)	Lipids and lipid-like molecules	Sphingolipids	0.90	0.05	0.85	1.24	0.01	0.31
Ceramide (d34:1)	Lipids and lipid-like molecules	Sphingolipids	-0.23	0.07	0.96	-0.32	0.01	0.42
Ceramide (d40:1)	Lipids and lipid-like molecules	Sphingolipids	0.47	<0.001	0.07	0.45	0.01	0.48
Ceramide (d34:2)	Lipids and lipid-like molecules	Sphingolipids	0.48	0.14	1.00	0.78	0.02	0.55
SM (d44:2)	Lipids and lipid-like molecules	Sphingolipids	-0.37	0.23	1.00	-0.71	0.02	0.55
SM (d30:1)	Lipids and lipid-like molecules	Sphingolipids	-0.15	0.12	1.00	-0.19	0.04	0.75
Ceramide (d42:0)	Lipids and lipid-like molecules	Sphingolipids	0.81	0.14	1.00	1.18	0.05	0.79
CE (18:3)	Lipids and lipid-like molecules	Steroids and steroid derivatives	-0.57	0.06	0.94	-0.76	0.01	0.42
Adenosine	Nucleosides, nucleotides, and analogues	Purine nucleosides	0.01	0.03	0.83	0.01	0.003	0.26
His-Ser	Organic acids and derivatives	Carboxylic acids and derivatives	0.37	0.02	0.77	0.49	0.001	0.26
Isoleucine	Organic acids and derivatives	Carboxylic acids and derivatives	-0.31	<0.001	0.06	-0.32	0.01	0.30
N-omega-Acetylhistamine	Organic acids and derivatives	Carboxylic acids and derivatives	-0.41	0.001	0.07	-0.44	0.02	0.49
Cocamidopropyl-Betaine	Organic acids and derivatives	Carboxylic acids and derivatives	-0.23	0.07	0.96	-0.29	0.03	0.60
Glutamine	Organic acids and derivatives	Carboxylic acids and derivatives	-0.62	0.10	1.00	-0.78	0.03	0.72
2-hydroxyglutaric acid	Organic acids and derivatives	Hydroxy acids and derivatives	0.48	0.01	0.70	0.64	0.001	0.26
1,5-Pentanediamine	Organic nitrogen compounds	Organonitrogen compounds	-1.17	<0.001	0.06	-1.18	0.003	0.26
SM (d42:3) A	Lipids and lipid-like molecules	Sphingolipids	0.25	0.001	0.07	0.24	0.02	0.49
SM (d42:3) B	Lipids and lipid-like molecules	Sphingolipids	0.97	0.001	0.10	0.95	0.03	0.60
Isothreonic acid	Organic oxygen compounds	Organooxygen compounds	-0.35	0.16	1.00	-0.53	0.04	0.72
Tryptophan	Organoheterocyclic compounds	Indoles and derivatives	-0.15	0.01	0.70	-0.20	0.001	0.26
5-methoxytryptamine	Organoheterocyclic compounds	Indoles and derivatives	-0.21	0.25	1.00	-0.46	0.02	0.52

[1] Multivariable linear regression analyses adjusted for age, race, parity, and prepregnancy BMI. Multiple comparisons were adjusted using the Benjamini-Hochberg procedure, with false discovery rates (FDRs) <0.05 as the statistically significant level.

Abbreviation: AC, acylcarnitines; BMI, body mass index; CE, cholesteryl esters; GWG, gestational weight gain; LPC, lysophosphatidylcolines; PC, phosphatidylcholine; PE, phosphatidylethanolamine; SM, sphingomyelin; TAG, triacylglycerol.

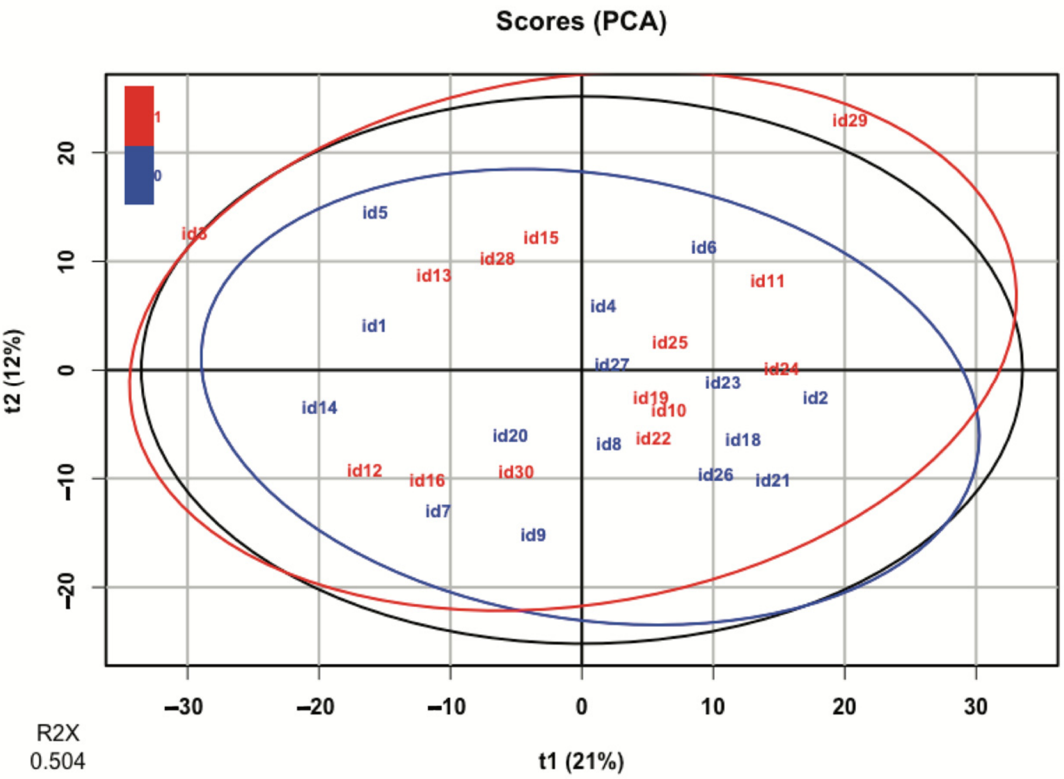

Figure S1. The principal component analysis score plot of the first two principal components of 29 pregnant women based on their metabolites collected around 32 weeks of gestation. The first component explained 21% of the variation, and the second component explained 12% of the variation. Mahalanobis distance-based confidence ellipses were used to represent intervention groups, with the red ellipse representing intervention group (i.e., lifestyle intervention) and the blue ellipse representing the control group (i.e., standard care).

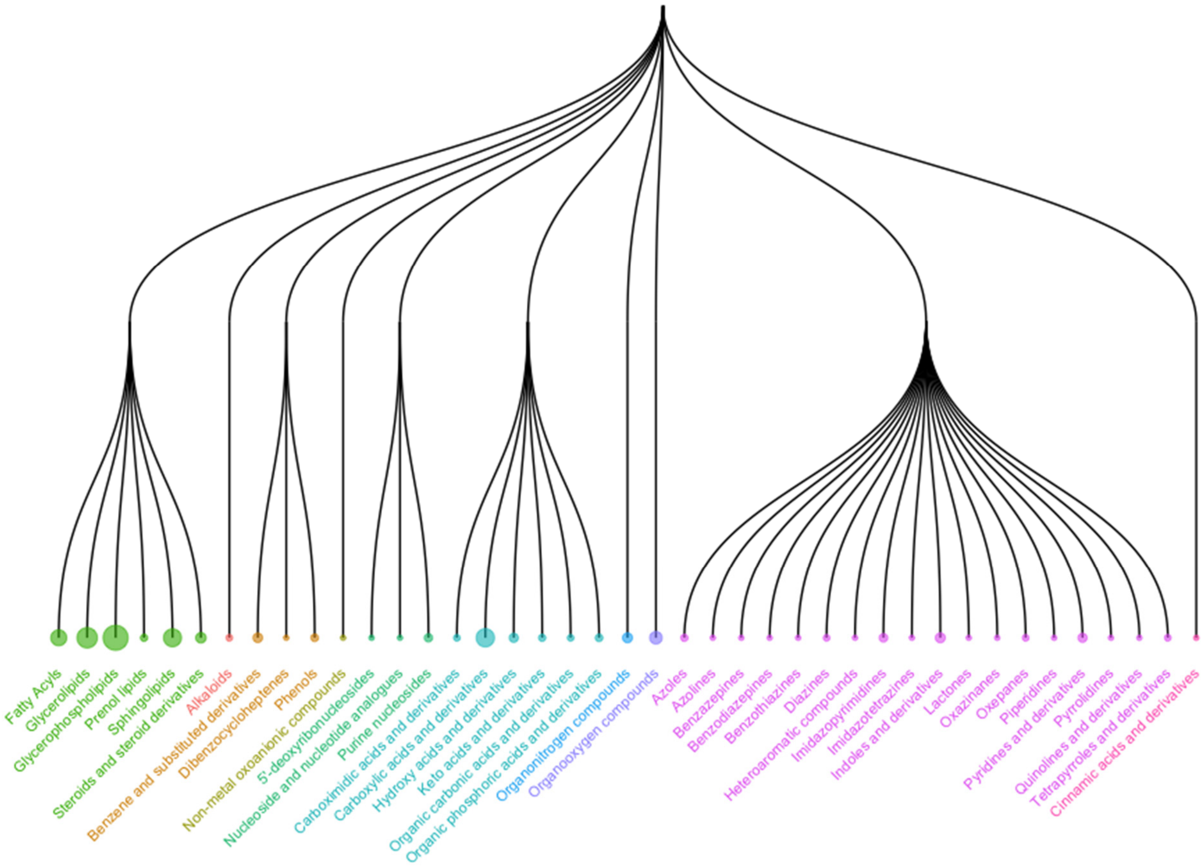

Figure S2. Dendrogram of the analyzed metabolites by superclass and class, for which the classification of chemical compound was performed using ClassyFire. From left to right, the superclasses are lipids and lipid-like molecules (in lime color), alkaloids and derivatives (in coral color), benzenoids (in pumpkin color), homogeneous non-metal compounds (in olive color), nucleosides, nucleotides, and analogues (in hunter color), organic acids and derivatives (in turquoise color), organic nitrogen compounds (in sea foam color), organic oxygen compounds (in periwinkle color), organoheterocyclic compounds (in plum color), phenylpropanoids and polyketides (in strawberry color), respectively. The node size denotes the number of metabolites in each class, which ranges from 1 to 223.

STROBE Statement—Checklist of items that should be included in reports of ***cohort studies***

	Item No	Recommendation	Page No
**Title and abstract**	1	(*a*) Indicate the study's design with a commonly used term in the title or the abstract	1
		(*b*) Provide in the abstract an informative and balanced summary of what was done and what was found	
**Introduction**			
Background/rationale	2	Explain the scientific background and rationale for the investigation being reported	1,2
Objectives	3	State specific objectives, including any prespecified hypotheses	2
**Methods**			
Study design	4	Present key elements of study design early in the paper	2
Setting	5	Describe the setting, locations, and relevant dates, including periods of recruitment, exposure, follow-up, and data collection	2
Participants	6	(*a*) Give the eligibility criteria, and the sources and methods of selection of participants. Describe methods of follow-up	2
		(*b*) For matched studies, give matching criteria and number of exposed and unexposed	
Variables	7	Clearly define all outcomes, exposures, predictors, potential confounders, and effect modifiers. Give diagnostic criteria, if applicable	2,3
Data sources/ measurement	8*	For each variable of interest, give sources of data and details of methods of assessment (measurement). Describe comparability of assessment methods if there is more than one group	2,3
Bias	9	Describe any efforts to address potential sources of bias	3,4
Study size	10	Explain how the study size was arrived at	2
Quantitative variables	11	Explain how quantitative variables were handled in the analyses. If applicable, describe which groupings were chosen and why	3,4
Statistical methods	12	(*a*) Describe all statistical methods, including those used to control for confounding	
		(*b*) Describe any methods used to examine subgroups and interactions	3,4
		(*c*) Explain how missing data were addressed	
		(*d*) If applicable, explain how loss to follow-up was addressed	
		(*e*) Describe any sensitivity analyses	
**Results**			
Participants	13*	(a) Report numbers of individuals at each stage of study—eg numbers potentially eligible, examined for eligibility, confirmed eligible, included in the study, completing follow-up, and analysed	4
		(b) Give reasons for non-participation at each stage	
		(c) Consider use of a flow diagram	
Descriptive data	14*	(a) Give characteristics of study participants (eg demographic, clinical, social) and information on exposures and potential confounders	
		(b) Indicate number of participants with missing data for each variable of interest	4
		(c) Summarise follow-up time (eg, average and total amount)	
Outcome data	15*	Report numbers of outcome events or summary measures over time	4
Main results	16	(*a*) Give unadjusted estimates and, if applicable, confounder-adjusted estimates and their precision (eg, 95% confidence interval). Make clear which confounders were adjusted for and why they were included	
		(*b*) Report category boundaries when continuous variables were categorized	4,5
		(*c*) If relevant, consider translating estimates of relative risk into absolute risk for a meaningful time period	
Other analyses	17	Report other analyses done—eg analyses of subgroups and interactions, and sensitivity analyses	4,5
**Discussion**			
Key results	18	Summarise key results with reference to study objectives	12

Limitations	19	Discuss limitations of the study, taking into account sources of potential bias or imprecision. Discuss both direction and magnitude of any potential bias	14
Interpretation	20	Give a cautious overall interpretation of results considering objectives, limitations, multiplicity of analyses, results from similar studies, and other relevant evidence	12,13, 14
Generalisability	21	Discuss the generalisability (external validity) of the study results	14
**Other information**			
Funding	22	Give the source of funding and the role of the funders for the present study and, if applicable, for the original study on which the present article is based	2,14

*Give information separately for exposed and unexposed groups.

Note: The Strengthening the Reporting of Observational Studies in Epidemiology (STROBE) Statement: guidelines for reporting observational studies (cohort studies). Available online: https://www.equator-network.org/reporting-guidelines/strobe/. (Accessed on July 20, 2022).
